# Mitochondrial ribosomal proteins: potential targets for cancer prognosis and therapy

**DOI:** 10.3389/fonc.2025.1586137

**Published:** 2025-04-30

**Authors:** Jianqing Zhu, Na Wen, Wen Chen, Haotian Yu

**Affiliations:** ^1^ Postgraduate Department, Hebei North University, Zhangjiakou, China; ^2^ Department of Obstetrics and Gynecology, The Eighth Medical Center, Chinese People's Liberation Army (PLA) General Hospital, Beijing, China; ^3^ Department of Pathology, The Eighth Medical Center, Chinese People's Liberation Army (PLA) General Hospital, Beijing, China

**Keywords:** mitochondrial ribosomes proteins, cancer, tumor microenvironment, prognostic marker, therapeutic target

## Abstract

Mitochondrial ribosomal proteins (MRPs) are essential components of mitochondrial ribosomes, responsible for translating proteins encoded by mitochondrial DNA and maintaining mitochondrial energy metabolism and function. Emerging evidence suggests that MRPs exhibit significant expression changes in multiple cancer types, profoundly affecting tumor biology through modulating oxidative stress levels, inducing metabolic reprogramming, disrupting cell cycle regulation, inhibiting apoptosis, promoting mitophagy, and remodeling the tumor microenvironment. Specifically, MRPs have been implicated in tumor cell proliferation, migration, invasion, and apoptosis, highlighting their potential as therapeutic targets. This review summarizes the multifaceted roles of MRPs in cancer, focusing on their impact on the tumor microenvironment and their potential as prognostic biomarkers and therapeutic targets. We also explore the implications of MRPs in precision oncology, particularly in patient stratification and the design of metabolic targeted therapies, offering new insights and research directions for the precise prevention and treatment of cancer.

## 1 Introduction

Cancer has become a major global health challenge, posing a serious threat to human life and well-being. The Global Cancer Observatory (GLOBOCAN) estimates that by 2022, there will be about 20 million new cancer cases and 9.7 million deaths annually (1). Among them, breast, lung, colorectal and stomach cancers are the types of cancers with high incidence and mortality rates (1). These statistics highlight the enormous burden of cancer control and emphasize the urgent need for effective strategies to reduce cancer incidence and mortality.

Cancer is a highly complex biological process involving the interaction of numerous internal and external factors. Tumor formation is closely linked to intracellular gene mutations and epigenetic alterations that contribute to the transformation of normal cells into malignant cells (2). The development of tumors depends on the transformation of cells and their interactions with the microenvironment (3), which involves major changes in cellular metabolism, signaling, and gene expression patterns (4). Cancer cells often exhibit changes in metabolic characteristics. For example, even in the presence of oxygen, glycolysis can increase (the Warburg effect), which gives them a growth advantage (5). This metabolic reprogramming is not only influenced by genetic factors, but is also closely related to hypoxic environments and mitochondrial dysfunction.

Mitochondria are the energy centers of the cell, responsible for producing ATP through oxidative phosphorylation (OXPHOS) and participating in key metabolic pathways (6). However, mitochondrial dysfunction is associated with a variety of diseases, including cancer (7). Mitochondrial dysfunction leads to increased oxidative stress, altered energy metabolism, and impaired cell signaling, all of which can contribute to the development of cancer. Recent studies have shown that mitochondrial dysfunction plays a key role in the carcinogenic process by affecting metabolic reprogramming, oxidative stress responses, and treatment responses (8).

Mitochondrial ribosomal proteins (MRPs) are crucial for maintaining mitochondrial function and synthesizing mitochondrial proteins (9). MRPs are encoded by nuclear genes and imported into the mitochondria, where they play an essential role in mitochondrial protein synthesis. New evidence suggests that the dysregulation of MRPs drives tumorigenesis by regulating tumor cell proliferation, migration, invasion, and apoptosis (10). MRPs can act as metabolic switches, linking mitochondrial dysfunction to cancer hallmarks such as sustained proliferation, evasion of apoptosis, and metastasis.

Given the critical role of MRPs in tumorigenesis and cancer progression, they offer new insights into precision oncology. They have the potential to serve as dual biomarkers for prognosis and therapeutic targeting. By linking mitochondrial dysfunction to cancer hallmarks, MRPs provide a unique opportunity to stratify patients based on their molecular profiles and design personalized therapies targeting cancer metabolism. This review aims to highlight the emerging role of MRPs in cancer research and explore their potential as prognostic markers and therapeutic targets to offer new insights and research directions for the precise prevention and treatment of cancer.

## 2 The functions of MRPs

Mammalian mitochondrial ribosomes consist of a small 28S subunit (mt-SSU) and a large 39S subunit (mt-LSU), which together form the 55S mitochondrial ribosome. The mt-SSU is assembled from 12S rRNA and 30 MRPs, while the mt-LSU is composed of 16S rRNA and 52 MRPs (11, 12). Mammalian mitochondrial ribosomes possess unique structural features that significantly distinguish them from bacterial ribosomes (13). For instance, the L1 stalk and P-site finger of mitochondrial ribosomes have distinct structures that are likely closely related to tRNA recognition and intersubunit communication (14). Additionally, the ratio of proteins to rRNA is higher in mammalian mitochondrial ribosomes, and this difference leads to substantial changes in their structure and function (15). For example, the reduced content of rRNA affects the structural characteristics of certain functionally relevant regions, such as tRNA binding sites and the exit tunnel for nascent polypeptides (14). Mammalian mitochondrial ribosomes contain at least 82 MRPs, 36 of which have no homologs in bacterial or cytoplasmic ribosomes (16). The presence of these unique MRPs, along with structural extensions of many MRPs that are homologous to bacterial ribosomal proteins, endows mammalian mitochondrial ribosomes with distinct functions in recognizing and translating mitochondrial mRNAs (16). Notably, a gate-like structural feature exists at the mRNA entrance of the mt-SSU, which is implicated in the recruitment of leaderless mitochondrial mRNAs to initiate protein synthesis on the mitochondrial ribosome (14). This feature reflects the adaptation of the mitochondrial ribosome to the characteristics of the mitochondrial genome during evolution and represents an important distinction of mammalian mitochondrial ribosomes from other ribosome types.

**Figure 1:**
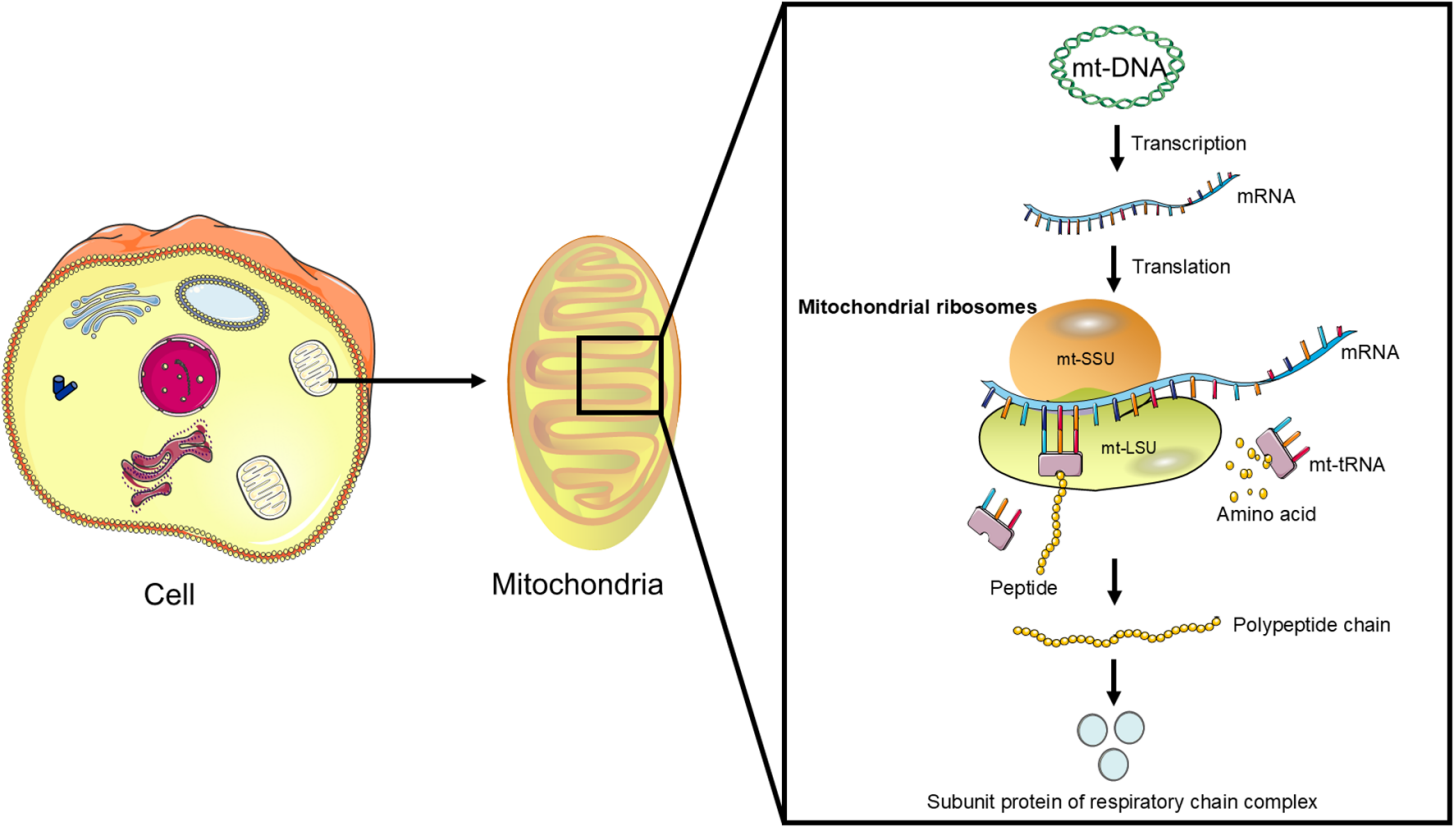
The function of mitochondrial ribosomes: Mitochondrial ribosomes consist of a small subunit (mt-SSU) and a large subunit (mt-LSU) that accurately translate mRNA transcribed from mitochondrial DNA (mt-DNA) into polypeptides, which subsequently assemble into subunit proteins of the respiratory chain complexes.

Mitochondrial ribosomes are responsible for translating the 13 proteins encoded by mitochondrial DNA (mt-DNA) (**Figure 1**), which are essential components of the mitochondrial respiratory chain complexes and crucial for mitochondrial energy metabolism and function (11). These proteins are involved in the process of OXPHOS, a key step in cellular energy production (12). The normal function of MRPs ensures efficient translation of mt-DNA-encoded proteins, thereby maintaining the integrity and functionality of the mitochondrial respiratory chain (17).

Recent studies have shown that some MRPs not only play important roles in protein synthesis within the mitochondria but also have biological functions outside the mitochondria. These functions involve the regulation of cell signaling, gene transcription, and associations with various diseases. For example, certain MRPs participate in the regulation of cell signaling pathways, affecting cell development and homeostasis. Abnormalities in MRPs may activate intracellular signaling pathways, such as the Notch1 and PI3K/AKT pathways (18, 19), which in turn can further influence cell proliferation, invasion, and metastasis. Moreover, MRPs regulate the transcription of mitochondrial genes through various mechanisms. For instance, *MRPL12* can directly bind to mitochondrial RNA polymerase (POLRMT) to regulate mitochondrial gene transcription (20). This regulatory mechanism is crucial for maintaining the balance of mitochondrial gene expression. Abnormalities in MRPs are associated with the development of various diseases. For example, mutations in the *MRPL12* gene can lead to decreased mitochondrial oxidative phosphorylation and impaired biosynthetic capacity, resulting in symptoms such as growth retardation, neurodegeneration, and defects in mitochondrial translation in patients (21). Additionally, abnormalities in MRPs are linked to the occurrence and development of cancer (10).

## 3 MRPs and cancer

The metabolic reprogramming of tumor cells is a key feature that enables them to adapt to rapid proliferation and harsh microenvironments (22). The Warburg effect, characterized by aerobic glycolysis, is a central manifestation of this reprogramming (5) and is closely related to the activation of oncogenes, inactivation of tumor suppressor genes, hypoxic conditions, mutations in mitochondrial DNA, and the genetic background of cancer cells (23). Despite these changes, OXPHOS remains important in some tumors, providing cancer cells with necessary energy (24). However, reduced OXPHOS function can decrease the production of reactive oxygen species (ROS), thereby inhibiting cancer cell growth and survival (24). MRPs play a crucial role in maintaining mitochondrial function and the operation of the OXPHOS system. Changes in their gene expression and function are closely related to OXPHOS damage, revealing the potential role of MRPs in cancer development and progression (25). Mitochondrial dysfunction is often accompanied by upregulation of MRP expression, which further promotes the shift of glucose metabolism towards glycolysis and lactate production (26), providing a growth advantage for cancer cells and being closely related to tumor molecular characteristics (27). In recent years, research has shown that the significant changes of MRPs in multiple cancers is closely associated with malignant proliferation, invasion, metastasis, and poor prognosis (**Table 1**). These findings suggest that their expression levels may serve as important biomarkers for tumor diagnosis, prognosis assessment, and treatment response. Therefore, in-depth research on the mechanisms of action of MRPs and their relationship with tumor metabolism is of great significance for revealing the molecular mechanisms of tumor development and progression and for developing new prognosis, diagnostic and therapeutic strategies.

**Table 1:** The association of MRPs with cancers.

Cancers	MRPs	Association of Transfer Traits with Expression Patterns	Expression	References
Breast cancer	MRPL3	Poor prognosis	↑	(31)
	MRPL12	Poor prognosis, proliferation, migration	↑	(32)
	MRPL13	Poor prognosis, proliferation, migration, EMT	↑	(29, 30, 32, 42–45)
	MRPL15	Tumor recurrence, distant metastasis, drug resistance		(146)
	MRPL41		↑	(156)
	MRPL52	Migration, invasion, EMT	↑	(18)
	MRPS6	Poor prognosis, Proliferation	↑	(49)
	MRPS18-2	Proliferation, metastasis, invasion		(34)
	MRPS23	Proliferation, metastasis	↑	(35, 47–49)
	MRPS27	Metastasis, drug resistance	↑	(46)
	MRPS30	Poor prognosis, proliferation, invasion	↑	(36–41)
Lung Cancer	MRPL9	Poor overall and relapse-free survival, metastasis	↑	(61)
	MRPL12	Poor prognosis, proliferation, migration, metastasis, invasion	↑	(54, 55, 57)
	MRPL13	Poor prognosis, proliferation, migration capacity, metastasis, invasion, EMT	↑	(52, 53)
	MRPL15	Poor prognosis	↑	(60)
	MRPL41		↓	(62)
LUAD	MRPL19	Poor prognosis, growth, migration, infestation	↑	(59)
	MRPL42	Poor prognosis, proliferation, migration, infestation	↑	(58)
Gastric Cancer	MRPL35	Poor prognosis, migration, infestation, transfer	↑	(72, 73)
	MRPL39	Poor prognosis,proliferation, migration, metastasis, invasion	↓	(76)
	MRPS5	Disease-specific survival	↓	(77)
	MRPS17	Poor prognosis, infestation	↑	(74, 75)
	DAP3 (MRPS29)	Poor prognosis, cell migration, drug resistance	↓	(78)
Liver cancer	MRPL13	Infestation		(25)
	MRPS31	Infestation		(86)
	MRPL38			(87)
	MRPS18A		↑	(81)
	MRPS23	Poor prognosis		(82, 83)
	MRPS5	Poor prognosis	↑	(84)
	MRPL48	Proliferation, migration, infestation		(85)
	MRPL9		↑	(88–90)
	MRPS12			(91, 92)
Colorectal cancer	MRPL52		↑	(68)
	MRPL33	Proliferation		(67)
	MRPL35	Proliferation	↑	(64)
	MRPL43	Poor prognosis, proliferation, infestation, migration	↑	(66)
	CRIF1 (MRPL64)		↓	(65)
	DAP3 (MRPS29)	Poor prognosis	↑	(70)
Thyroid cancer	MRPL44	Lymphatic node transfer		(97)
	MRPL9	Proliferation, migration	↑	(95)
	MRPL14	Proliferation, migration, invasion	↑	(96)
SACC	MRPL23	Poor prognosis, metastasis	↑	(98)
OSCC	MRPL23		↓	(99)
	MRPL52		↑	(100)
OPSCC	MRPL33		↑	(101)
HNSCC	MRPL34		↑	(101)
	MRPL47		↑	(102)
Head and neck tumor	MRPL11		↑	(94)
AML	MRPL49		↑	(107)
	CRIF1 (MRPL64)		↓	(105, 106)
	MRPL33		↑	(108)
ALL	MRPL47			(157)
Glioma	MRPL42	Proliferation	↑	(113)
	MRPS16	Proliferation, migration, infestation	↑	(19)
Glioblastoma	MRPL35			(114)
Ovarian cancer	MRPL15	Poor prognosis	↑	(116)
	MRPS31		↓	(116)
	MRPS12	Poor prognosis	↑	(117)
Cervical cancer	MRPL11	Proliferation, infestation		(119)
Endometrial cancer	MRPS18-2	Proliferation	↑	(118)
Neuroblastoma	MRPL3	Event-free survival		(127)
Cholangiocarcinoma	MRPS18A	Poor prognosis		(128)
	MRPL27	Poor prognosis	↑	(129)
Pancreatic cancer	MRPL28		↓	(123)
	MRPL12		↓	(123)
Prostate cancer	MRPS18-2	Migration	↑	(122)
Bladder cancer	MRPL4			(125)
	MRPL23	Poor prognosis	↑	(124)
Uveal melanoma	MRPS11			(130)
Renal cancer	MRPL41		↓	(62)
ACC	MRPS23	Proliferation	↓	(121)
Osteosarcoma	MRPS7			(126)

↑, Increased expression of MRPs is associated with the trait. ↓, Decreased expression of MRPs is associated with the trait. LUAD, lung adenocarcinoma. SACC, salivary adenoid cystic carcinoma. OSCC, oral squamous cell carcinoma. OPSCC, oropharyngeal squamous cell carcinoma. HNSCC, head and neck squamous cell carcinoma. AML, acute myeloid leukemia. ALL, acute lymphoblastic leukemia. ACC, adrenocortical carci-noma. EMT, epithelial-mesenchymal transition.

### 3.1 MRPs and breast cancer

Breast cancer is one of the most common malignancies among women worldwide, characterized by high rates of incidence and mortality (28). Recent studies have highlighted the crucial role of MRPs in the occurrence, development, and prognosis of breast cancer (29), suggesting a strong correlation between mitochondrial function and tumor cell behavior. Specifically, MRPs are involved in key cellular processes such as cell proliferation, apoptosis, and cell cycle regulation (30), all of which directly influence breast cancer formation and progression.

Multiple studies have shown that the expression of several MRP genes is significantly upregulated in breast cancer cells and tissues, such as *MRPS30*, *MRPL3*, *MRPL12*, *MRPL13*, *MRPL52*, *MRPS6*, *MRPS18–2* and *MRPS23* (18, 31–36). This upregulation underscores the close link between mitochondrial function and the proliferation and survival of breast cancer cells. *MRPS30* plays a key role in the development of breast cancer, particularly in disrupting cell behavior (37). The high levels of MRPS30-DT in breast cancer patients are positively correlated with poor prognosis, and knocking down MRPS30-DT in the breast cancer cell significantly inhibits cell proliferation and invasion and induces apoptosis (38), suggesting its potential as a prognostic biomarker and therapeutic target. Genome-wide association studies (GWAS) have identified several genetic variants within the *MRPS30* gene on chromosome 5p12, such as rs930395, rs10941679, rs2067980, and rs4415084, which are positively correlated with increased breast cancer risk (39, 40). The risk allele of rs4415084 is activated by coordinating the function of the *MRPS30* gene, further confirming its importance in breast cancer development (39, 40). Another study found that the single nucleotide polymorphism (SNP) rs7716600 and rs4415084-T risk allele on chromosome 5p12 region is positively correlated with high expression levels of *MRPS30* and its neighboring long non-coding RNA RP11-53O19.1 (36, 41). The rs4321755-T allele promotes the transcription of *MRPS30* and RP11-53O19.1 through an enhancer within a GATA3 binding site, leading to changes in GATA3 binding and chromatin accessibility (41). The *MRPS30* gene in 5p12 regio is upregulated in estrogen receptor-positive(ER-positive) breast cancer, suggesting that this SNP may influence the development of estrogen receptor-positive tumors by regulating the expression of *MRPS30* (36). Luciferase reporter gene assays have shown that the functional SNP rs3747479 (*MRPS30*) significantly alters the promoter activity of the target gene in both ER-positive and ER-negative breast cancer cell lines (37), further validating the functional role of *MRPS30* and its genetic variants in breast cancer.

**Figure 2:**
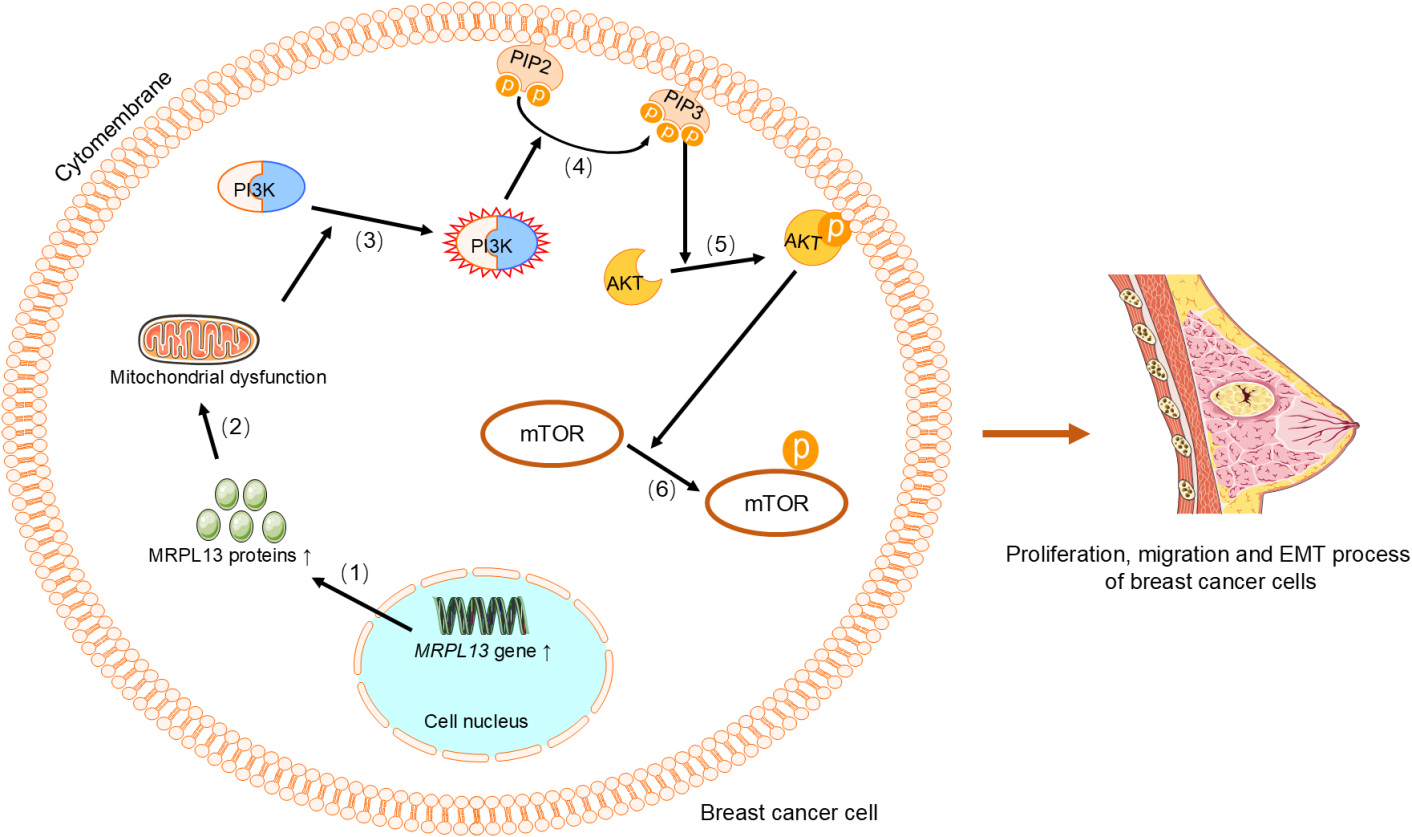
MRPL13 regulates the PI3K/AKT/mTOR signaling pathway to promote proliferation, migration, and EMT in breast cancer cells. (1) Upregulation of the *MRPL13* gene increases the synthesis of MRPL13 protein, (2) which in turn causes mitochondrial dysfunction. (3) This alteration in mitochondrial function aberrantly activates PI3K, (4) which phosphorylates PIP2 on the cell membrane to generate PIP3. (5) PIP3 recruits and activates AKT, (6) and phosphorylated AKT subsequently activates mTOR. Phosphorylated mTOR promotes proliferation and migration of breast cancer cells, as well as the EMT process. PI3K, Phosphoinositide 3-Kinase. PIP2, phosphatidylinositol-4,5-bisphosphate. PIP3, phosphatidylinositol-3,4,5-trisphosphate. AKT, Protein Kinase B. mTOR, Mechanistic Target of Rapamycin. EMT, epithelial-mesenchymal transition.

**Figure 3:**
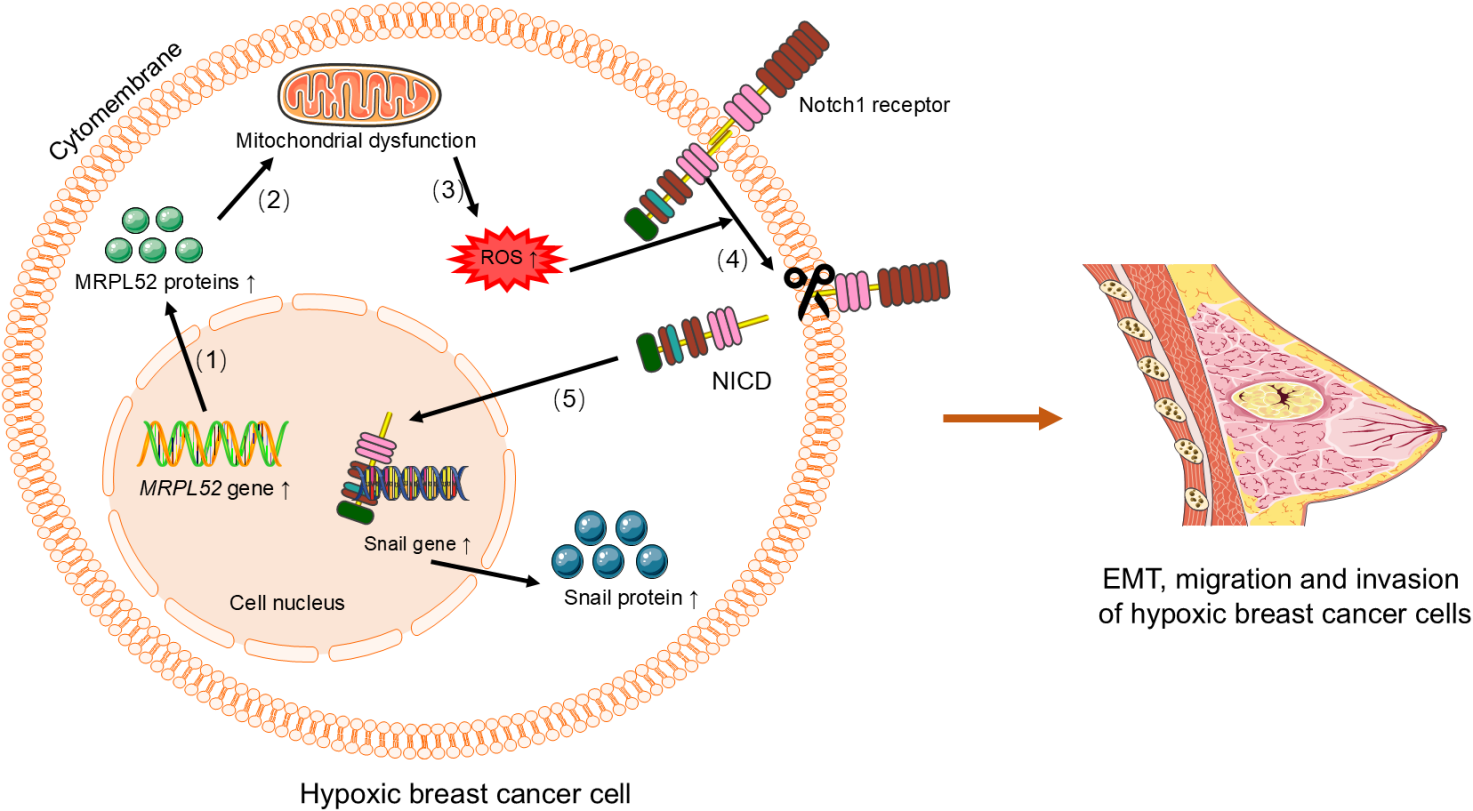
MRPL52 regulates the ROS/Notch1/Snail signaling pathway to promote EMT, migration, and invasion in hypoxic breast cancer cells. (1) Upregulation of *MRPL52* expression leads to increased synthesis of MRPL52 protein. (2) The increased MRPL52 protein causes mitochondrial dysfunction. (3) Mitochondrial dysfunction induces increased production of ROS. (4) ROS activates the Notch1 receptor, which is cleaved to release the NICD. (5) NICD translocates to the nucleus and activates the expression of Snail gene, leading to increased Snail protein synthesis. The Snail protein promotes EMT, migration, and invasion in hypoxic breast cancer cells. EMT, epithelial-mesenchymal transition. ROS, reactive oxygen species. NICD, Notch intracellular domain.

The expression of the *MRPL3* is upregulated in breast tumor tissues, positively correlating with cancer progression, receiver operating characteristic(ROC) curve and Kaplan-Meier survival analyses, which indicates its potential as a diagnostic and prognostic biomarker for breast cancer (31). Similarly, *MRPL13* promotes the growth and spread of breast cancer by influencing cellular metabolism and energy demands, and its high expression is significantly correlated with clinical pathological factors and considered a poor prognostic indicator (29, 30, 32, 42–44). Functional studies have shown that MRPL13 promotes proliferation and migration as well as epithelial-mesenchymal transition (EMT) process by triggering activation of the PI3K/AKT/mTOR signaling pathway in breast cancer cells (**Figure 2**) (45). This highlights its significant role in breast cancer metastasis and invasion. Similarly, knocking down *MRPL12* and *MRPL13 in vitro* significantly inhibits breast cancer cell activity and migration (32). The expression of *MRPL52* is upregulated in breast cancer, particularly in hypoxic breast cancer cells, which regulates the ROS/Notch1/Snail signaling pathway to promote EMT, migration, and invasion (**Figure 3**), which enhances apoptotic resistance and metastatic potential (18).

In triple-negative breast cancer (TNBC), MRPS27 expression is closely related to tumor staging and lymph node involvement, and reducing its expression can effectively inhibit the stem cell properties and malignant phenotypes of TNBC, which highlights its potential as a therapeutic target (46). Similarly, the phosphorylated form of MRPS23 by mitogen-activated protein kinase may be involved in the proliferation of breast cancer cells (35). The amplification of *MRPS23* is positively correlated with high proliferation rates and the emergence of non-basal subtypes in breast cancer (47). The overexpression of *MRPS6* and *MRPS23* in breast cancer cells and tissues is associated with increased tumor cell proliferation. Knocking down these two genes inhibits cell proliferation, reduces the expression of oncogenes and mesenchymal markers, and promotes the expression of tumor suppressor genes (48, 49). Immunohistochemical analysis also shows that the expression of MRPS18–2 is significantly higher in high-proliferation tumors (such as luminal A and basal types) than in low-proliferation tumors, indicating that MRPS18–2 may be a potential marker for the aggressiveness of breast cancer (34).

In summary, specific SNPs located in the 5p12 region, such as rs7716600 and rs930395, are closely associated with breast cancer susceptibility and high expression of MRP genes. These findings suggest that genetic variation in MRPs may be one of the genetic bases for breast cancer susceptibility. Moreover, MRPs not only participate in tumor proliferation and invasion through upregulated gene expression but also regulate mitochondrial function, cellular metabolism, and cell signaling through various mechanisms, thereby promoting the malignant transformation of breast cancer. High MRP expression is typically closely related to tumor aggressiveness and poor prognosis.

### 3.2 MRPs and lung cancer

Lung cancer is one of the most common malignant tumors globally and the leading cause of cancer-related deaths (50). Due to the lack of obvious early symptoms and effective early diagnostic methods, many patients are diagnosed with advanced lung cancer (51). In recent years, research has found that MRPs play a significant role in the occurrence, development, and drug resistance of lung cancer, providing new insights for the diagnosis and treatment of this disease.

Multiple studies have shown that the expression levels of several MRPs are significantly upregulated in lung cancer tissues and are closely related to tumor proliferation, invasion, and drug resistance. For example, *MRPL13* is highly expressed in lung adenocarcinoma (LUAD) and is positively correlated with biological processes such as proliferation, invasion, DNA repair, cell cycle progression, EMT, and metastasis (52). Additionally, the expression of *MRPL13* is negatively correlated with hypoxia and inflammation modules, further supporting its role in the tumor microenvironment (52). Gene set enrichment analysis (GSEA) results suggest that *MRPL13* may participate in the development and progression of lung cancer by modulating key signaling pathways such as MYC targets, PI3K/AKT/mTOR signaling, oxidative phosphorylation, and the G2/M checkpoint (53). Experimental findings also indicate that the expression of *MRPL13* is significantly higher in lung cancer tissues than in normal tissues. Knocking down *MRPL13* in LUAD significantly reduces cancer cell viability, delays tumor division and migration, diminishes invasive capacity, and promotes apoptosis (52). Therefore, *MRPL13* not only holds potential value for the diagnosis of LUAD but may also serve as a prognostic biomarker for certain malignancies.

*MRPL12* is upregulated in LUAD and promotes tumor progression by enhancing mitochondrial OXPHOS. Studies have shown that *MRPL12* is highly expressed in human LUAD tissues, mouse LUAD tissues driven by Tp53fl/fl and KrasG12D, LUAD patient-derived organoids (PDO), and LUAD cell lines, correlating with poor patient survival (54–57). Overexpression of *MRPL12* significantly promotes LUAD tumorigenesis, metastasis, and PDO formation, while *MRPL12* knockdown elicits the opposite phenotype (54–57). Additionally, YTHDC2 has been identified as a factor that inhibits the proliferation, invasion, and migration of LUAD cells by binding to m6A-modified *MRPL12* mRNA and disrupting its stability, thereby promoting apoptosis. However, the upregulation of *MRPL12* expression attenuates the inhibitory effect of YTHDC2 on cancer cell proliferation (54, 56, 57). Furthermore, research has shown that the phosphorylation of MRPL12 at the Y60 site is crucial for its oncogenic functions. The dephosphorylation of MRPL12 Y60 by UBASH3B inhibits the binding of MRPL12 to POLRMT, thereby downregulating mitochondrial metabolism in LUAD cells. In-depth *in vivo*, *in vitro*, and organoid model validations have confirmed that the *MRPL12* Y60 mutation significantly inhibits LUAD progression (55). In summary, *MRPL12* acts as a novel oncogene in LUAD, promoting tumor development through mitochondrial metabolism reprogramming towards OXPHOS.

The upregulation of *MRPL42* in early-stage LUAD tissues and cell lines is significantly associated with patient prognosis (58). Knocking down *MRPL42* not only reduces the proliferation and colony-forming ability of LUAD cells but also leads to cell cycle arrest at the G1/S phase, thereby inhibiting cell migration and invasion. *In vivo* experiments have also confirmed that the absence of *MRPL42* significantly suppresses tumor growth (58).Bioinformatics analysis suggests that the transcription factor YY1 may promote the transcription of the *MRPL42* gene by binding to its upstream promoter region (58). This finding has been validated by chromatin immunoprecipitation (ChIP) and dual-luciferase reporter assays (58). Further qRT-PCR experiments show that knocking down YY1 significantly reduces the expression level of *MRPL42* (58). This indicates that *MRPL42* acts as an oncogene in LUAD, with its expression regulated by YY1.The expression of *MRPL19* is upregulated in LUAD and is associated with lymph node metastasis, tumor differentiation, and pathological status, indicating poor prognosis (59). Functional network analysis suggests that *MRPL19* may be regulated by multiple miRNAs and the E2F family and is involved in processes such as the cell cycle, cell adhesion molecules, spliceosome, and T-helper cell differentiation (59). GSEA and protein-protein interaction (PPI) network analysis indicate that *MRPL19* is closely related to lung cancer proliferation signaling pathways (59). *In vitro* experiments have shown that knocking down *MRPL19* significantly inhibits the growth, migration, and invasion of LUAD cells, further confirming its oncogenic role in LUAD (59).

In non-small cell lung cancer (NSCLC), the expression of *MRPL15* is significantly upregulated and is closely associated with gender, clinical stage, lymph node status, and TP53 mutation status. Patients with high expression of *MRPL15* have poorer overall survival (OS), progression-free survival (PFS), disease-free survival (DFS), and recurrence-free survival (RFS) (60). These results suggest that *MRPL15* may serve as a potential prognostic biomarker for NSCLC. Further functional network analysis indicates that *MRPL15* is involved in metabolic-related pathways, DNA replication, and cell cycle processes through various signaling pathways involving kinases, miRNAs, and transcription factors (60). Immunohistochemical (IHC) experiments further validate the high expression of MRPL15 in NSCLC and confirm its potential as a prognostic marker (60).The expression of *MRPL9* is upregulated in lung cancer tissues and is associated with OS and RFS in patients (61). Knocking down *MRPL9* significantly reduces the proliferation, colony formation, and migration capabilities of lung cancer cells. Further research indicates that *MRPL9* may promote lung cancer progression by regulating the transcription of c-MYC, especially in lung cancer tissues with high c-MYC expression (61). In A549 cells, the expression of *MRPL9* remains unchanged after c-MYC is knocked down, suggesting that *MRPL9* may promote lung cancer metastasis by regulating c-MYC transcription, thereby affecting the expression of migration-related molecules (61).

In contrast, *MRPL41* is expressed at lower levels in various small cell lung cancer cell lines, and its downregulation is associated with tumor progression. Studies have shown that *MRPL41* enhances the stability of p53 and promotes apoptosis induced by p53 under growth-inhibitory conditions such as actinomycin D treatment and serum starvation (62). This suggests that *MRPL41* may act as a tumor suppressor in lung cancer, and its downregulation could be one of the important mechanisms for tumor malignant transformation.

### 3.3 MRPs and colorectal cancer

Colorectal cancer is one of the most common malignant tumors globally and is the second leading cause of cancer-related deaths (63). In recent years, studies have found that multiple MRPs play important roles in the occurrence, development, and metastasis of colorectal cancer. *MRPL35* is highly expressed in colorectal cancer tissues, and its high expression is significantly associated with shortened OS in patients (64). *In vitro* experiments have shown that downregulation of *MRPL35* significantly increases the generation of ROS, leading to DNA damage, inhibiting cell proliferation, and triggering G2/M phase arrest (64). Additionally, downregulation of *MRPL35* reduces mitochondrial membrane potential and induces apoptosis and autophagy. In a colorectal cancer xenograft model in nude mice, knockdown of *MRPL35* effectively inhibits tumor proliferation (64). These results suggest that *MRPL35* may play a significant role in the development and progression of colorectal cancer by regulating ROS generation and cell cycle progression. *CRIF1* (also known as *MRPL64*), recruited to the promoter region of the p53 gene, was initially identified as an interacting protein of Gadd45γ and can inhibit cell growth and tumor formation by interacting with p53 and Gadd45 family proteins, and was also found to be associated with good prognosis in colorectal cancer, suggesting its potential as a tumor suppresso (65).

The expression of *MRPL43* is significantly increased in colorectal cancer tissues. Bioinformatics analysis has revealed that the rs4919510 variant G allele in miR-608 is associated with high expression of *MRPL43*, and knockdown of the *MRPL43* gene effectively inhibits the proliferation, invasion, and migration capabilities of the colorectal cancer HCT-116 cell line and promotes apoptosis (66), suggesting that the rs4919510 variant G allele in miR-608 may influence the occurrence and progression of colorectal cancer by upregulating the expression of *MRPL43*. The absence of the long isoform of MRPL33 (MRPL33-L) results in impaired cell proliferation and increased apoptosis. Further analysis has found that the deficiency of MRPL33-L is accompanied by mitochondrial dysfunction, including the accumulation of ROS, reduced ATP generation, and decreased levels of 16S rRNA (67). In human colorectal cancer tissues, the expression of *MRPL33* containing exon 3 is upregulated, and its expression level is highly correlated with that of hnRNPK. This suggests that hnRNPK may play a significant role in tumorigenesis by regulating the splicing of *MRPL33* pre-mRNA (67).Through proteomics analysis and public transcriptomics data, *MRPL52* has been identified as a key protein in colon cancer cells. Its expression level is significantly higher in colon tumor samples than in normal colon samples (68). Gene dependency analysis shows that silencing the expression of *MRPL52* effectively inhibits the proliferation of colon cancer cells (68). Moreover, the characteristics of the *MRPL52* gene may significantly predict the survival of colorectal cancer patients, with significant prognostic performance in both the training and test sets (69). In colorectal cancer tissues, the transcription and protein levels of *DAP3* (also known as *MRPS29*) and *DELE1* are significantly higher than those in normal tissues. Their expression levels are closely related to the clinical outcomes and local recurrence of patients, suggesting that they may serve as potential prognostic biomarkers (70).

### 3.4 MRPs and gastric cancer

Gastric cancer is the fifth most common cancer globally and is often diagnosed at an advanced stage, resulting in a poor prognosis (71). Due to the difficulty in early clinical detection and the poor outcomes of late-stage treatments, there is an urgent need to identify new tumor biomarkers and therapeutic targets to aid in the prevention, diagnosis, and treatment of gastric cancer (72, 73). The expression of *MRPL35* is significantly upregulated in gastric cancer tissues and is closely associated with patient age, lymph node metastasis, and pathological tumor-node-metastasis (TNM) staging, both *in vivo* and *in vitro* experiments have shown that knocking down *MRPL35* significantly inhibits the proliferation and colony formation of gastric cancer cells and induces apoptosis (72). This indicates that *MRPL35* may play a significant role in the development and progression of gastric cancer, with its high expression potentially enhancing the proliferation and anti-apoptotic capabilities of gastric cancer cells, thereby affecting disease progression and prognosis. Additionally, the expression levels of *MRPS17* are also typically higher in gastric cancer tissues than in normal tissues. A follow-up study of 100 gastric cancer patients showed that patients with positive *MRPS17* expression had a significantly worse prognosis than those with negative expression, and high MRPS17 expression, as an independent prognostic factor, was closely related to the aggressiveness of gastric cancer (74). Another study also demonstrated that high expression levels of MRPS17 are significantly associated with OS and RFS in patients with gastric adenocarcinoma (75). This suggests that MRPS17 may influence the progression of gastric cancer by affecting cellular behaviors such as proliferation and invasion, with its high expression potentially indicating a greater likelihood of the disease progressing towards more aggressive and metastatic directions, thus having an adverse impact on patient survival. Conversely, the expression of *MRPL39* is significantly downregulated in gastric cancer tissues and cell lines, and low expression is closely associated with clinical features of the tumor, especially tumor size and TNM staging. Gastric cancer patients with low levels of *MRPL39* expression have significantly shorter OS and DFS (76). Further functional validation experiments have shown that overexpression of *MRPL39* significantly inhibits the growth, proliferation, migration, and invasion of gastric cancer cell lines BGC823 and SGC-7901 (76). Based on qRT-PCR analysis of data from cancerous and adjacent normal tissues in the GTEx and TCGA databases, immunohistochemistry data from the Human Protein Atlas (HPA) database, and analysis of clinical surgical samples from gastric cancer patients, the expression levels of *MRPS5* in gastric cancer are significantly lower than those in adjacent normal tissues (77). Research has found that high expression of *DAP3* in gastric cancer is associated with better prognosis, suggesting its potential as a prognostic biomarker. Additionally, knocking down the expression of *DAP3* can promote cell migration by inhibiting apoptosis (78). This suggests that the roles of *MRPL39*,*MRPS5*,and *DAP3* in gastric cancer may be complex. Their high expression may, to some extent, inhibit the malignant progression of tumors, while their downregulation may weaken the apoptotic capacity of cells, thereby promoting the survival and migratory ability of tumor cells and influencing the invasiveness and metastatic potential of gastric cancer.

### 3.5 MRPs and liver cancer

Liver cancer is one of the most common malignant tumors globally, with a complex pathogenesis and significant biological heterogeneity. These characteristics pose substantial challenges for treatment and are major reasons for treatment failure and recurrence (79). Recent studies have shown that mitochondria play a key role in apoptosis and metabolism, and their dysfunction may be associated with cancer development (80). Changes in the expression of MRPs in liver cancer are closely related to cancer development and progression. Different MRP genes influence the biological characteristics of liver cancer and patient prognosis by regulating mitochondrial function, metabolic pathways, and cellular invasion activity. *MRPL13*-mediated defects in OXPHOS were found to enhance hepatoma cell invasiveness by increasing the expression of claudin-1 (CLN1) (25). *MRPS18A* has been identified as a downstream target of miR-514a-5p, with its expression increased in hepatocellular carcinoma cells. Recent studies have shown that upregulation of *MRPS18A* can restore the inhibitory effects of TRIM52-AS1 downregulation on the biological functions of hepatocellular carcinoma cells (81). Moreover, the expression of *MRPS18A* is closely related to the prognosis of hepatocellular carcinoma patients, with high expression potentially indicating a worse prognosis (81). *In vitro* and *in vivo* experiments indicate that high expression of *MRPS23* in hepatocellular carcinoma is associated with low patient survival rates (82, 83). High expression of the Sirtuin-1 and *MRPS5* enhances metabolic flexibility and is associated with poor prognosis in patients with hepatocellular carcinoma (84). Bioinformatic analysis and *in vitro* model studies show that knockdown of *MRPL48* reduces the proliferation, migration, and invasion of hepatocellular carcinoma cells (85). This suggests that *MRPL48* may play a significant role in the development of hepatocellular carcinoma, with its high expression potentially promoting the malignant behavior of hepatocellular carcinoma cells. However, inhibition of *MRPS31* can lead to mitochondrial dysfunction, thereby enhancing the invasiveness of hepatocellular carcinoma cells (86).This suggests that *MRPS31* may play a balancing role in maintaining mitochondrial function and cellular invasiveness in hepatocellular carcinoma cells. Additionally, several genetic association studies have shown significant correlations with liver cancer. Whole-exome sequencing (WES) results from four subjects revealed that the C.430G>C (p.Gly144Arg) variant in the *MRPL38* gene is a key genetic factor for hepatocellular carcinoma (87). This provides genetic evidence for the potential role of *MRPL38* in liver cancer, with its variation potentially affecting the risk of liver cancer development. *MRPL9* is a protein-coding gene involved in mitochondrial translation, which is most closely related to mRNA stemness index (mRNAsi) in hepatocellular carcinoma tissues and is significantly overexpressed in hepatocellular carcinoma patients, making it a potential prognostic biomarker for hepatocellular carcinoma (88–90). *MRPS12* is associated with the prognosis of HBV-related hepatocellular carcinoma and drives the malignant phenotype of hepatocellular carcinoma by regulating mitochondrial metabolism (91, 92). This indicates that *MRPS12* may play a significant role in the metabolic reprogramming and disease progression of liver cancer.

### 3.6 MRPs and head and neck cancer

Head and neck cancer refers to malignant tumors that occur in the head and neck region, including cancers of the thyroid, salivary glands, oral cavity, and larynx (93). The development of many head and neck cancers is closely related to mitochondrial dysfunction, especially in energy metabolism and apoptosis (94). Therefore, mutations in mt-DNA that encode OXPHOS proteins are considered to have an important association with the occurrence of human head and neck cancer.

In the study of thyroid cancer, the overexpression of several MRP genes is closely related to the occurrence and development of tumors. For example, *MRPL9* is overexpressed in papillary thyroid carcinoma (PTC) cells, significantly promoting cell proliferation and migration capabilities, while its knockdown inhibits these abilities. PTC cells with MRPL9 gene knocked out were transplanted subcutaneously into nude mice, which not only inhibits the growth of subcutaneous xenograft tumors in nude mice but also significantly reduces the incidence of lung metastasis (95), indicating that *MRPL9* may serve as a potential biomarker for PTC. *MRPL14* also exhibits significant oncogenic effects in thyroid cancer. Its overexpression is closely related to advanced tumor stages, extrathyroidal invasion, and lymph node metastasis (96). *MRPL14* accelerates the proliferation and migration of thyroid cancer cells by promoting the expression of EMT-related proteins (96). Conversely, the knockdown of *MRPL14* leads to decreased expression of mitochondrial respiratory chain complex IV (MTCO1) and increased intracellular ROS levels. When cells are co-treated with ROS scavengers, their proliferation and migration capabilities are restored, further confirming the key role of ROS in the oncogenic mechanism of *MRPL14* (96). Additionally, the mRNA expression level of *MRPL44* is closely related to the OXPHOS metabolic phenotype in PTC and is significantly associated with lymph node metastasis (97). Based on OXPHOS levels, the expression of *MRPL44* may serve as a representative biomarker of the metabolic phenotype and has the potential to become an important biomarker for predicting lateral neck lymph node metastasis in thyroid cancer patients (97).

In salivary gland adenoid cystic carcinoma (SACC), the high expression of MRPL23-AS1 is significantly associated with pulmonary metastasis and OS in patients. MRPL23-AS1 promotes the EMT process by forming an RNA-protein complex with Enhancer of Zeste Homolog 2 (EZH2), which enhances the binding of EZH2 to the H3K27me3 mark on the E-cadherin promoter, thereby promoting cell migration and invasion (98). Furthermore, MRPL23-AS1 not only functions within tumor cells but also influences tumor metastasis through exosomes. MRPL23-AS1-containing exosomes interact with pulmonary microvascular endothelial cells, affecting microvascular permeability and thereby facilitating the metastasis of SACC (98). However, the expression pattern of MRPL23-AS1 is not consistent across different tumors. For example, in oral squamous cell carcinoma (OSCC), MRPL23-AS1 is expressed at lower levels compared to normal tissues (99). Conversely, *MRPL52* is significantly upregulated in OSCC, and Kaplan-Meier survival analysis shows that patients with high *MRPL52* expression have significantly worse PFS and DSS, indicating that *MRPL52* may serve as a poor prognostic biomarker for OSCC (100).

In human papillomavirus (HPV)-positive oropharyngeal squamous cell carcinoma and HPV-negative head and neck squamous cell carcinoma (SCCHN), *MRPL33* is abnormally overexpressed and shows significant differential expression (101). Furthermore, high expression of MRPL47 is closely related to a high-risk score in patients with SCCHN. This risk score is significantly associated with patient prognosis, indicating the potential of MRPL47 as a prognostic indicator (102).

### 3.7 MRPs and leukemia

Leukemia is a malignant tumor of the hematopoietic system, characterized by the abnormal proliferation of leukemia cells. Mitochondria, as key organelles for cellular energy metabolism and biosynthesis, play an important role in the occurrence and development of leukemia (103). MRPs are responsible for synthesizing proteins encoded by the mitochondrial genome, which are core components of the electron transport chain and OXPHOS, essential for maintaining normal cellular function (104). Therefore, mitochondrial dysfunction may directly impact the proliferation and survival of leukemia cells.

The role of *CRIF1* in leukemia has attracted the attention of researchers. Studies have found that the mRNA and protein expression of *CRIF1* are significantly reduced in patients with acute myeloid leukemia (AML), a phenomenon that may be associated with the uncontrolled proliferation of leukemia cells (105). Further experiments indicate that *CRIF1* overexpression induces G0/G1 phase cell cycle arrest in Jurkat cells, while its depletion reduces this arrest, highlighting the crucial role of CRIF1in cell cycle regulation (105). Additionally, *CRIF1* depletion can reverse the cell cycle arrest induced by bone marrow stromal cells in leukemia cells, further confirming the importance of *CRIF1* in regulating leukemia cell growth (105). Immunoprecipitation experiments have revealed that CRIF1 specifically binds to CDK2 during cell cycle arrest, suggesting that CRIF1 may control the cell cycle process by regulating CDK2 activity (105). Notably, CRIF1 is also one of the interacting proteins of Lymphocyte-specific protein tyrosine kinase (Lck). Immunofluorescence microscopy and immunoprecipitation experiments have further confirmed the association between CRIF1 and Lck in the nucleus, where Lck promotes cell survival and may inhibit the activity of CRIF1 through its interaction. Silencing of CRIF1 also promotes the survival of leukemia T cells in the absence of growth factors, further validating its role in leukemia cell survival (106).

In addition to the role of CRIF1 in leukemia, other MRPs have also been implicated in the progression of leukemia. For example, *MRPL49* is identified as a potential target gene in AML. The upregulation of miRNAs, including hsa-mir-520a, 599, 606, 137, and 362, may increase the prognostic risk for AML patients by regulating the expression of *MRPL49* (107). Another study demonstrated that *MRPL33* expression is elevated in AML cell lines and correlates with receptor tyrosine kinase expression, such as TrkA or KIT, suggesting *MRPL33* as a potential prognostic biomarker for AML (108).

### 3.8 MRPs and glioma

**Figure 4:**
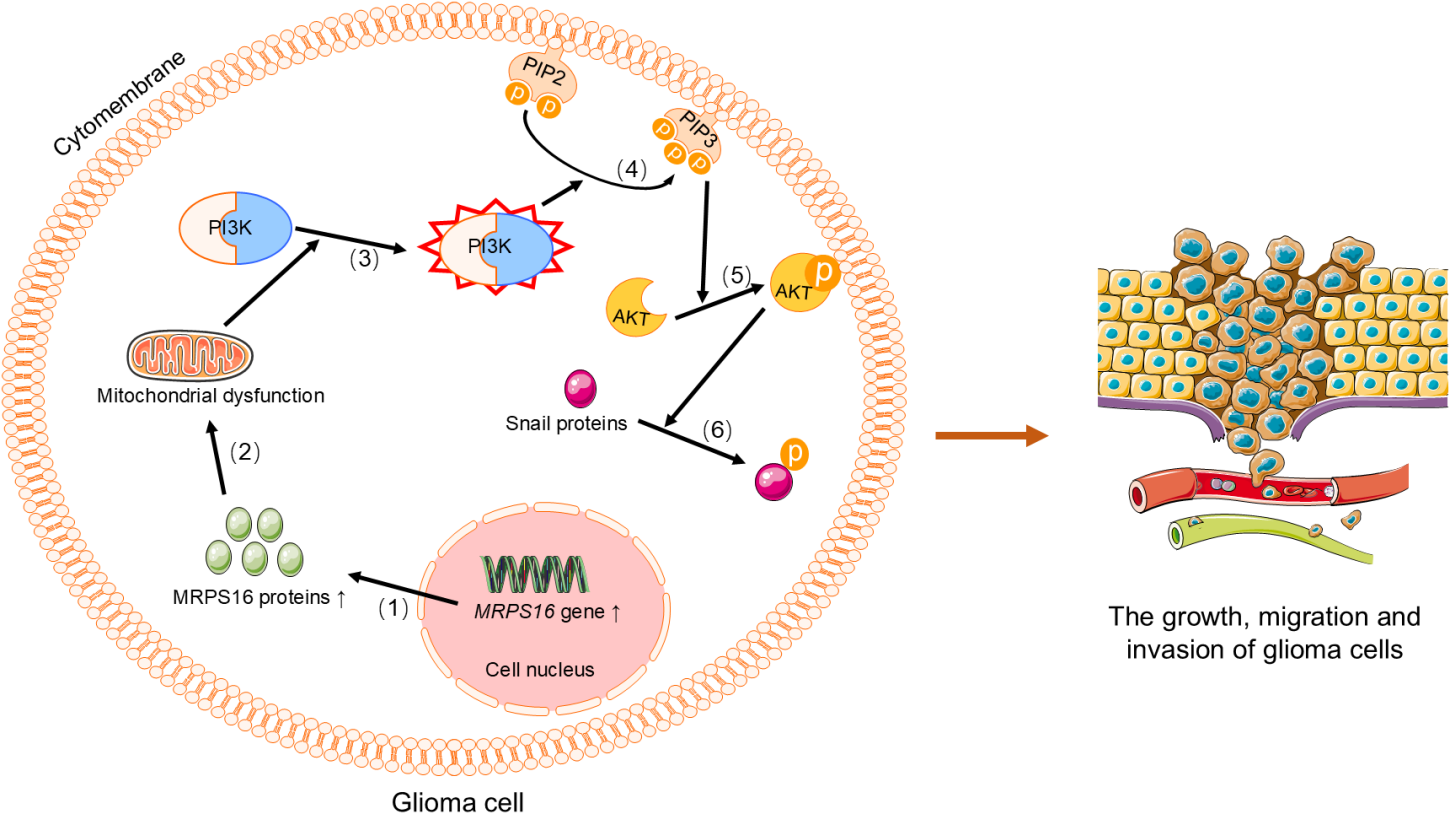
MRPS16 regulates the PI3K/AKT/Snail signaling axis to promote the growth, metastasis, and invasion of glioma cells. (1) Upregulation of *MRPS16* leads to increased synthesis of MRPS16 protein.(2) MRPS16 protein activates PI3K, (3) which PIP2 on the cell membrane to generate PIP3, (4) PIP3 recruits and activates AKT, (5) and phosphorylated AKT activates Snail protein. Phosphorylated Snail protein promotes the growth, migration, and invasion of glioma cells. PI3K, Phosphoinositide 3-Kinase. PIP2, phosphatidylinositol-4,5-bisphosphate. PIP3, phosphatidylinositol-3,4,5-trisphosphate. AKT, Protein Kinase B.

Gliomas are brain tumors that originate from glial cells in the central nervous system, with the most common and aggressive type being glioblastoma multiforme (109, 110). Glioma cells are characterized by rapid proliferation and invasion, and they exhibit significant metabolic reprogramming (111). This metabolic shift confers a growth advantage on tumor cells. Moreover, mitochondria in glioma cells not only contribute to energy metabolism but also play a crucial role in regulating cellular metabolism and the oxidative stress response (112). By analyzing the TCGA database, researchers have found that *MRPL42* is significantly upregulated in glioma tissues (113). Further studies have shown that knocking down *MRPL42* significantly inhibits the proliferation of U251 and A172 glioma cells, while activating apoptosis and caspase 3/7 activity (113). This suggests that *MRPL42* may play an important role in the development of glioma. Additionally, silencing *MRPL42* leads to an increase in cell cycle distribution at the G1 and G2/M phases, and a decrease in the S phase, further confirming its key role in tumor cell growth (113). Therefore, *MRPL42* is considered a potential oncogene in glioma. Similarly, the expression of *MRPS16* in glioma has also garnered significant attention. Through Western blot, qRT-PCR, and IHC analyses, researchers have found that *MRPS16* expression is significantly increased in tumor tissues compared to normal brain tissues, especially in high-grade gliomas. Knockdown experiments of *MRPS16* have shown that it can inhibit tumor cell growth, migration, and invasion, while overexpression of *MRPS16* enhances these tumor characteristics (19). Additionally, silencing *MRPS16* significantly reduces the expression levels of proteins such as Snail, p-AKT, and p-PI3K in U-138MG and U-87MG cells (19), further indicating that *MRPS16* promotes glioma cell proliferation and invasion through the PI3K/AKT/Snail signaling axis (**Figure 4**). These findings suggest that *MRPS16* may serve as a potential prognostic biomarker for glioma. In recent years, other mitochondrial ribosomal proteins, such as MRPL30, MRPL35, MRPS17, MRPL20, MRPS31, MRPL58, and MRPL40, have also been identified as novel biomarkers in the pathogenesis of glioblastoma (114).

### 3.9 MRPs and female genital tumors

Female genital tumors, including ovarian cancer, endometrial cancer, and cervical cancer, pose a significant threat to women’s health (115). Recent studies have found that MRPs are closely related to the occurrence and development of these tumors and may serve as potential diagnostic and prognostic biomarkers. In ovarian cancer, six MRP genes (*MRPL10*, *MRPL15*, *MRPL36*, *MRPL39*, *MRPS16*, and *MRPS31*) were identified as related to ovarian cancer through the STRING database, and exhibit significant differences in expression patterns in ovarian cancer tissues, with *MRPL10*, *MRPL15*, *MRPL36*, *MRPL39*, and *MRPS16* being significantly upregulated, while *MRPS31* is downregulated (116). Kaplan-Meier survival analysis shows that high expression of *MRPL15*, *MRPL36*, *MRPL39*, and *MRPS16* is significantly associated with poorer OS in ovarian cancer patients (116). Notably, *MRPL15* has the highest expression level in ovarian cancer and is closely related to cell cycle, DNA repair, and mTOR1 signaling pathways, potentially promoting the occurrence and development of ovarian cancer through gene amplification and hypomethylation (116). In addition to the aforementioned MRP genes, the overexpression of *MRPS12* in ovarian cancer has also garnered significant attention from researchers. Analysis using the Oncomine and GEPIA databases has shown that overexpression of *MRPS12* is associated with poorer OS in ovarian cancer patients, especially in those with advanced stages (III+IV), serous ovarian cancer, and TP53 mutations (117). These findings suggest that *MRPS12* may function as an oncogene in ovarian cancer and has the potential to serve as a prognostic biomarker. In endometrial cancer, the expression level of *MRPS18–2* is elevated and closely correlated with high expression of *E2F1*. Both *in vitro* and *in vivo* experiments have further demonstrated that overexpression of *MRPS18–2* significantly promotes the proliferation of endometrial cancer cells (118). Moreover, overexpression of *MRPS18–2* in endometrial cancer cells is associated with decreased signaling of pan-keratin, β-catenin, and E-cadherin, while showing increased vimentin signaling (118).In cervical cancer, univariate analysis has shown that the expression of *MRPL11* and *MRPS23* is significantly associated with PFS, and high expression of these genes is closely related to rapid proliferation of cervical cancer cells, oxidative phosphorylation, invasiveness, and tumor size, indicating that they may play important roles in the progression and metastasis of cervical cancer (119).

### 3.10 MRPs and other cancers

In other cancers, MRPs also exhibit significant roles. In adrenal adenomas, *CRIF1* expression is significantly reduced compared to adjacent normal tissues, suggesting its involvement in tumor suppression through negative regulation of cell cycle and growth pathways (120). Similarly, *MRPS23* is downregulated in metastatic adrenocortical carcinoma and correlates with patient survival, highlighting its potential as a prognostic biomarker (121). In renal cancer, *MRPL41*, stabilizing p53, is downregulated in cancer cell lines compared to pre-cancerous cell lines and promotes its translocation to the mitochondria, inducing apoptosis., and leading to cell cycle arrest at the G1 phase in the absence of p53 (62), which suggests that *MRPL41* downregulation may contribute to renal cancer progression. In prostate cancer, *MRPS18–2* expression increases with disease progression, which promotes EMT via the TWIST2/E-cadherin pathway, enhancing cell migration (122). In pancreatic cancer, *MRPL28* and *MRPL12* downregulation promotes tumor growth in cell lines such as SU86 and Miapaca2 (123). In bladder cancer, high expression of MRPL23-AS1 is associated with poor prognosis (124), while *MRPL4* expression correlates with overall survival in patients with muscle-invasive bladder cancer treated with cisplatin-based neoadjuvant chemotherapy (125). In osteosarcoma, *MRPS7* is considered a potential biomarker (126), and in neuroblastoma, lnc-MRPL3–2 expression predicts event-free survival (127). In cholangiocarcinoma, high expression of *MRPS18A* and *MRPL27* is associated with poor prognosis and shortened survival and with clinical staging, histological grading, and Child-Pugh classification, indicating its role in disease progression (128, 129). In uveal melanoma, *MRPS11* expression serves as a prognostic predictor (130). These findings further underscore the diverse roles of MRPs in promoting cancer progression through various molecular mechanisms, emphasizing their broad impact on cancer biology.

## 4 MRPs and the tumor microenvironment

The tumor microenvironment (TME) is a complex extracellular environment surrounding cancer cells, comprising the extracellular matrix (ECM), blood vessels, immune cells, and non-cancerous host cells (131). Historically, these components were considered bystanders in tumorigenesis. However, recent studies have shown that they play a crucial role in tumor initiation, progression, and metastasis (131). The cellular composition and functional status of the TME are influenced by multiple factors, including the organ in which the tumor is located, the characteristics of cancer cells, the tumor stage, and individual patient differences (131). In the early stages of tumor growth, cancer cells dynamically interact with TME components to support their survival, invasion, and metastasis (132, 133). Specifically, cancer cells recruit and reprogram non-cancerous host cells, reshape the vascular system and ECM, and create a supportive environment for tumor growth (131). To overcome the hypoxic and acidic conditions within the tumor, the TME induces angiogenesis to restore oxygen and nutrient supply while clearing metabolic waste (132). Meanwhile, immune cells within the TME exhibit both pro-tumor and anti-tumor effects, influencing tumor growth and metastasis (132).

Mitochondria, as the energy metabolism hubs of cells, are closely related to the formation and maintenance of the TME (134). MRPs play a crucial role in maintaining mitochondrial function and regulating cellular metabolism. They not only directly contribute to the proliferation of tumor cells but also further influence tumor progression through interactions with the TME. Mitochondria regulate the functions of immune cells through various mechanisms, including metabolic pathways, amino acid metabolism, antioxidant systems, mitochondrial dynamics, mt-DNA, mitophagy, and mitochondrial reactive oxygen species (mtROS) (135). These mechanisms play crucial roles in the activation, differentiation, and survival of immune cells, thereby directly influencing immune function (135). Mitochondrial translation is essential for synthesizing mitochondrially encoded proteins, and inhibiting mitochondrial translation can severely impact the function of immune cells. For example, certain antibiotics targeting mitochondrial ribosomes, such as linezolid, have been shown to have strong immunosuppressive effects (136). This immunosuppression is linked to the inhibition of mitochondrial translation in Th17 cells, leading to mitonuclear imbalance, loss of mitochondrial activity, and reduced cytokine production (136). These findings indicate that MRPs are crucial for maintaining the functional integrity of immune cells, and disrupting them can have profound effects on immune responses.

Moreover, mitochondrial dysfunction can negatively affect the immune system. For instance, abnormal mitochondrial function can regulate the expression of PD-L1, and high PD-L1 expression can, in turn, feedback and affect mitochondrial metabolism, thereby influencing immune evasion and tumor progression (137). Mitochondrial dysfunction also leads to significant upregulation of immune cell infiltration, inflammatory responses, and ECM-related gene expression, which may create a tumor-promoting microenvironment by suppressing normal immune responses and facilitating immune evasion (138).Additionally, mitochondrial dysfunction can upregulate the expression of lactate dehydrogenase (LDH), further promoting aerobic glycolysis, which not only provides energy support for tumor cells but also acidifies the tumor microenvironment, creating favorable conditions for tumor cell growth (139).

MRPs interact with the TME, influencing tumor biology through metabolic reprogramming and regulation of cellular functions within the TME. Abnormal expression of MRPs can disrupt immune cell function, leading to tumor immune evasion. For example, the expression of *MRPL13* is associated with immune infiltration patterns in multiple cancers and is negatively correlated with M1 macrophages, CD8+ T cells, and CD4+ T cells (52, 53). CIBERSORT analysis shows that higher *MRPL13* expression in breast cancer is significantly associated with reduced NK cell infiltration (43). Similarly, *MRPL15* expression in NSCLC is negatively correlated with immune infiltration, including immune and stromal scores and tumor-infiltrating lymphocytes (TILs) (60). In ovarian cancer, high expression of *MRPL15* is closely related to the proliferation of CD8 T cells and dendritic cells, as well as the expression of TGFβR1 and IDO1 (116).Another study on ovarian cancer indicates that the expression of *MRPS12* is positively correlated with the infiltration of macrophages and neutrophils (117). Additionally, the expression of *MRPL12* in LUAD is significantly associated with immune regulatory factors, chemokines, and the infiltration levels of various immune cells (57), suggesting its potential role in the tumor immune microenvironment. In functional network analysis, *MRPL19* may be associated with the differentiation process of T helper cells, and immune infiltration analysis shows that the expression of *MRPL19* is closely related to the infiltration of B cells, CD4 T cells, and dendritic cells in LUAD, which may impact disease progression (59). In gastric adenocarcinoma, the expression of *MRPS17* is significantly negatively correlated with the abundance of TILs, indicating that high expression of *MRPS17* may reduce immune cell infiltration and further influence immune evasion mechanisms (75). Additionally, high expression of *MRPL47* is considered a potential biomarker for immune suppression in patients with SCCHN (102).

In summary, MRPs significantly influence immune evasion and tumor progression through complex interactions with the TME. Specifically, MRPs alter immune infiltration patterns within the tumor microenvironment by regulating immune cell functions and metabolic reprogramming, thereby promoting the growth and metastasis of tumor cells.

## 5 The therapeutic potential of MRPs in cancer

As research into the role of MRPs in tumor initiation, progression, and drug resistance mechanisms deepens, their potential as therapeutic targets in cancer is gradually emerging. MRPs play crucial roles in processes such as metabolic reprogramming, cell cycle regulation, apoptosis inhibition, and immune evasion in tumor cells. These multifaceted functions make them highly promising therapeutic targets.

### 5.1 MRP-targeted drugs

In recent years, researchers have discovered that various antibiotics can inhibit tumor cell proliferation by targeting mitochondrial protein synthesis. For example, tetracycline antibiotics (such as doxycycline and tigecycline) bind to the mitochondrial small subunit to inhibit mitochondrial protein synthesis, thereby suppressing the metabolic function of tumor cells (140). Studies have shown that doxycycline not only inhibits tumor cell proliferation but also induces apoptosis and has demonstrated good anti-tumor effects in various tumor models (141, 142). Additionally, erythromycin and chloramphenicol inhibit mitochondrial protein synthesis by blocking peptide bond formation or the peptide exit tunnel, thereby suppressing tumor cell metabolism and proliferation (143).

However, long-term use of these antibiotics may lead to side effects, such as inhibiting cellular energy metabolism and causing cell proliferation arrest (141). Therefore, the development of MRP-targeted drugs with higher specificity and fewer side effects has become a research hotspot. For example, potassium tellurate (K_2_TeO_3_), a strong oxidizing agent, inhibits tumor cell growth and metabolism by damaging mitochondrial ribosomal function (144). Moreover, some studies have found that certain compounds (such as acetaminophen, carbamazepine, and tunicamycin) can exert anti-tumor effects by targeting specific MRPs (such as MRPL12 and MRPL13) (32). These findings highlight the potential of developing novel therapeutic strategies targeting MRPs to enhance tumor treatment efficacy while minimizing adverse effects.

### 5.2 MRPs in tumor drug resistance and immunotherapy

The mechanisms underlying the role of MRPs in tumor drug resistance primarily involve metabolic reprogramming and the regulation of signaling pathways. For example, in breast cancer, high expression of *MRPL15* is closely related to tamoxifen resistance. Studies have shown that inhibiting the expression of *MRPL15* can re-sensitize tumor cells to the drug (145, 146). Similarly, overexpression of *MRPS23* in breast cancer cells enhances resistance to CDK1 inhibitors, while knocking down *MRPS23* using shRNA technology restores drug sensitivity (35). These findings suggest that targeting MRPs can serve as an effective strategy for reversing drug resistance.

Moreover, tumor mutation burden (TMB) is significantly correlated with the expression levels of *MRPL13*. Abnormal expression of *MRPL13* can affect TMB levels, which in turn impacts the response to tumor immunotherapy. Therefore, *MRPL13* not only serves as a biomarker for predicting treatment response in breast cancer patients but also provides a new target for evaluating and optimizing immunotherapy (30). These results highlight the potential of *MRPL13* as a valuable indicator for guiding personalized treatment strategies and improving therapeutic outcomes in cancer patients.

The role of MRPs in the tumor immune microenvironment offers new insights for immunotherapy. Studies have shown that abnormal expression of MRPs can influence immune infiltration patterns in tumors, thereby affecting their ability to evade the immune system. For example, *MRPL13* is closely related to immune cell infiltration in various cancers, with its high expression negatively correlated with the infiltration of M1 macrophages, CD8^+^ T cells, and CD4^+^ T cells (52, 53). Additionally, the expression of *MRPL19* in LUAD is closely associated with the infiltration levels of B cells, CD4 T cells, and dendritic cells (59). These findings suggest that modulating the expression of MRPs can alter the tumor immune microenvironment, enhancing immune cell infiltration and anti-tumor activity. Moreover, some studies have found that targeting MRPs can enhance the efficacy of immune checkpoint inhibitors. For example, in a breast cancer model, knocking down the expression of MRPS30-DT using shRNA technology significantly inhibited tumor cell proliferation and invasion and enhanced the cytotoxic effect of immune cells on tumors (38). This indicates that MRPs can serve not only as potential targets for immunotherapy but also as a means to improve treatment outcomes when combined with other immunotherapeutic approaches.

In summary, MRPs hold broad application prospects in cancer therapy. By delving into their mechanisms of action in tumor development, drug resistance, and the immune microenvironment, it is hoped that more precise and effective cancer treatment strategies can be developed, offering new breakthroughs for clinical treatment.

## 6 The limitations and challenges of MRP-targeted therapies in cancer

As essential components of the mitochondrial translation system, MRPs are involved in the translation of mt-DNA and regulation of energy metabolism (10). In recent years, MRPs have garnered increasing attention as therapeutic targets due to their significant roles in cancer. However, there are multiple limitations and challenges in clinical MRP-Targeted Therapies in cancer. Firstly, the heterogeneity of tumors complicates treatment. Tumors are typically composed of multiple clones, and targeting multiple clones is more likely to reduce the likelihood of drug resistance due to clonal selection, thereby improving overall patient response (147, 148). Moreover, the expression and function of MRPs vary significantly across different tumors. For instance, MRPL12 is significantly downregulated in pancreatic cancer (123), but significantly upregulated in breast and lung cancers (32, 55). This tissue specificity, coupled with differences in mitochondrial metabolism among various cell types, further complicates precision targeting. Secondly, the high membrane potential and the double-membrane structure of mitochondria limit the penetration of traditional drugs (149). Although positively charged targeting units such as triphenylphosphonium ions are widely used, they are prone to non-specific binding with proteins or enzymes, leading to drug aggregation and reduced targeting efficiency (150). Additionally, as MRPs are primarily located in the mitochondrial inner membrane or matrix (151), drugs need to cross multiple barriers to reach their target sites. Thirdly, the multifunctionality of MRPs increases the risk of off-target effects. MRPs are involved not only in mitochondrial protein translation but also in the regulation of functions such as OXPHOS (10). For example, MRPL12 activates mt-DNA transcription through its interaction with POLRMT, and its abnormality can affect mitochondrial translation and energy metabolism (152). This multifunctionality means that targeting interventions may trigger widespread metabolic disorders or cell apoptosis, resulting in serious off-target effects. MRP-targeted therapies represent a promising target in preclinical research. However, various obstacles hinder the effective clinical translation of these approaches. In the process of clinical translation, the lack of biomarkers and individual heterogeneity make it difficult to precisely identify patient populations that are sensitive to mitochondrial-targeted therapies (153). Additionally, there may be some potential toxicity in MRP-targeted therapies. Drug delivery systems used for these therapies, such as lipid nanoparticles (LNPs), may cause safety issues, including inflammation, immunogenicity, and cytotoxicity (154). Some MRP-targeted drugs may excessively inhibit mitochondrial function, affecting healthy tissues (104), and even interfere with immune cell functions, impacting the immune cell infiltration in the tumor microenvironment (52). MRP-targeted therapies may also have cumulative toxicity, with toxic reactions potentially worsening as treatment duration extends (155). Many MRP-targeted therapies are still in the research phase, lacking long-term clinical safety data, which makes it difficult to fully assess their potential risks in clinical practice. Lastly, the specific mechanisms by which MRPs influence cancer progression and treatment response have not been fully elucidated, and insufficient target validation limits the development of MRP-targeted therapies.

## 7 Conclusions and future research directions

**Figure 5:**
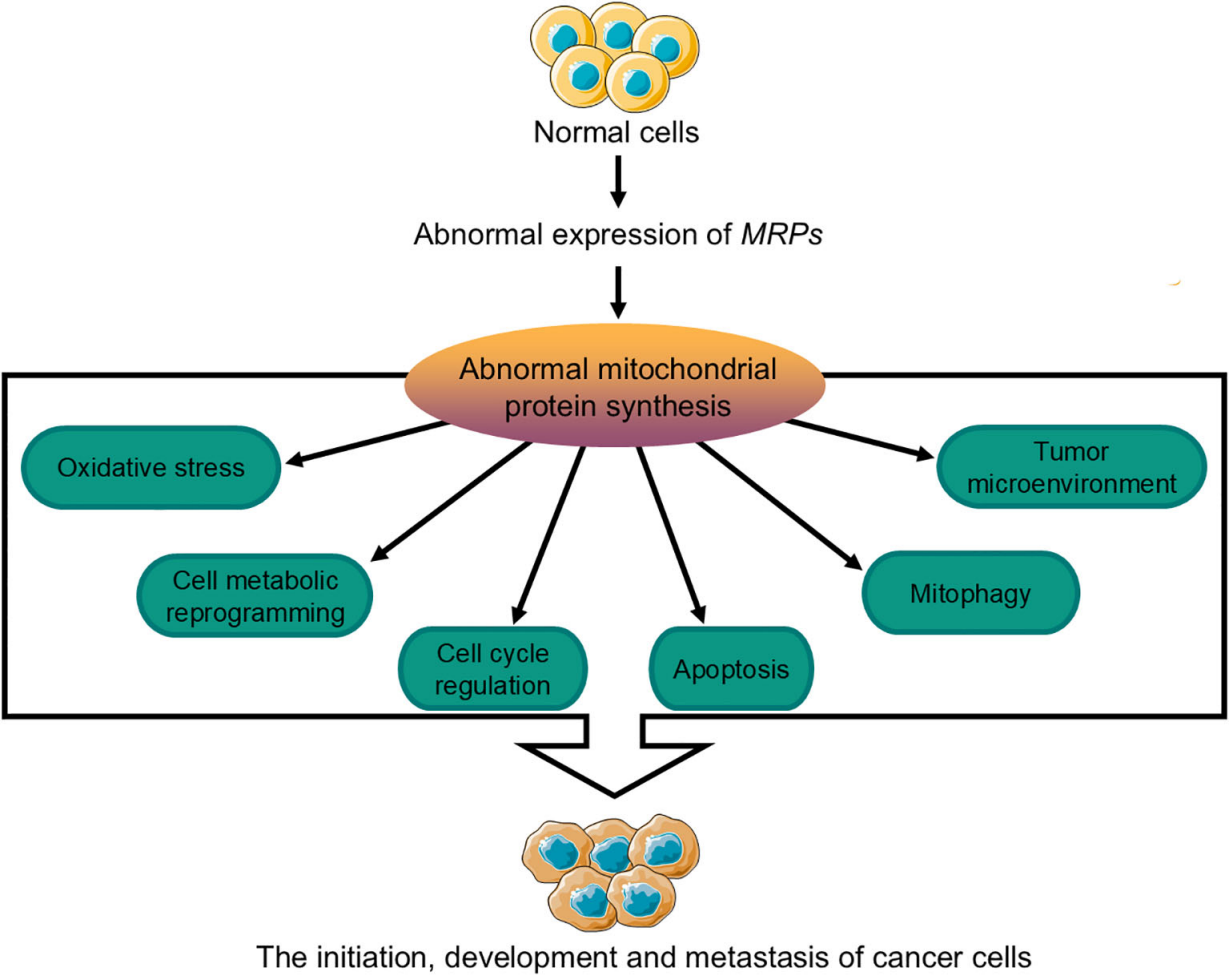
Mechanisms by which MRPs influence the initiation, progression, and metastasis of cancer cells. The abnormal expression of *MRPs* can affect the synthesis of mitochondrial proteins in normal cells, which may lead to tumor initiation, development, and metastasis through mechanisms involving changes in cellular oxidative stress levels, metabolic reprogramming, cell cycle regulation, apoptosis, mitophagy, and the tumor microenvironment. MRPs, mitochondrial ribosomal proteins.

Mitochondrial dysfunction, as a central aspect of cancer metabolism, has positioned MRPs as key regulators of this process. Aberrant expression of MRPs impairs mitochondrial ribosome function and leads to abnormal mitochondrial protein synthesis. These impairments drive tumor initiation, progression, metastasis, and immune evasion by modulating oxidative stress levels, inducing metabolic reprogramming, disrupting cell cycle regulation, inhibiting apoptosis, promoting mitophagy, and remodeling the tumor microenvironment (**Figure 5**). Moreover, changes in MRPs expression levels across different cancer types, such as breast, lung, gastric, liver, and colorectal cancers, have significant implications. These changes not only serve as potential biomarkers for early diagnosis but also provide valuable targets for prognostic assessment and personalized treatment strategies. By understanding the role of MRPs in cancer metabolism, researchers can develop more effective diagnostic tools and therapeutic interventions to improve patient outcomes.

There is still considerable progress to be made in improving MRP-targeted therapies and translating these findings into effective clinical applications that can more efficiently achieve therapeutic goals in cancer. In the future, it will be essential to investigate the specific mechanisms of MRPs in tumorigenesis in greater depth, accumulate definitive clinical evidence, and develop effective cancer therapies based on MRPs. Moreover, focusing on the development of combination therapy strategies—such as integrating MRP-targeted treatment with chemotherapy or immunotherapy—may significantly enhance treatment outcomes by leveraging synergistic effects or overcoming drug resistance. Furthermore, the discovery and validation of biomarkers are critical for advancing personalized treatment approaches and identifying patient populations most likely to benefit from MRP-targeted therapies. This includes exploring the potential of MRPs as predictive biomarkers for immunotherapy response. Lastly, optimizing drug delivery systems to enhance mitochondrial penetration—such as designing lipid nanoparticles (LNPs) with improved mitochondrial targeting efficiency—and identifying MRP-specific inhibitors with minimal off-target effects are vital steps in improving treatment efficacy and reducing potential toxicity. Future efforts should integrate technological innovations with interdisciplinary research, while gathering further evidence from both basic and clinical studies to ensure precision and safety in MRP-targeted therapies.

## References

[1] Bray F Laversanne M Sung H Ferlay J Siegel RL Soerjomataram I. Global cancer statistics 2022: GLOBOCAN estimates of incidence and mortality worldwide for 36 cancers in 185 countries. CA Cancer J Clin. (2024) 74:229–63. doi: 10.3322/caac.21834 38572751

[2] Yang Y Wang Y. Role of epigenetic regulation in plasticity of tumor immune microenvironment. Front Immunol. (2021) 12:640369. doi: 10.3389/fimmu.2021.640369 33868269 PMC8051582

[3] Hanahan D. Hallmarks of cancer: new dimensions. Cancer Discovery. (2022) 12:31–46. doi: 10.1158/2159-8290.CD-21-1059 35022204

[4] You M Xie Z Zhang N Zhang Y Xiao D Liu S. Signaling pathways in cancer metabolism: mechanisms and therapeutic targets. Signal Transduct Target Ther. (2023) 8:196. doi: 10.1038/s41392-023-01442-3 37164974 PMC10172373

[5] Akter R Awais M Boopathi V Ahn JC Yang DC Kang SC. Inversion of the Warburg effect: unraveling the metabolic nexus between obesity and cancer. ACS Pharmacol Transl Sci. (2024) 7:560–9. doi: 10.1021/acsptsci.3c00301 PMC10928896 38481689

[6] Ryu KW Fung TS Baker DC Saoi M Park J Febres-Aldana CA. Cellular ATP demand creates metabolically distinct subpopulations of mitochondria. Nature. (2024) 635:746–54. doi: 10.1038/s41586-024-08146-w PMC11869630 39506109

[7] Annesley SJ Fisher PR. Mitochondria in health and disease. Cells. (2019) 8:680. doi: 10.3390/cells8070680 31284394 PMC6678092

[8] Kampen KR Sulima SO Vereecke S De Keersmaecker K. Hallmarks of ribosomopathies. Nucleic Acids Res. (2020) 48:1013–28. doi: 10.1093/nar/gkz637 PMC7026650 31350888

[9] Cheong A Lingutla R Mager J. Expression analysis of mammalian mitochondrial ribosomal protein genes. Gene Expr Patterns. (2020) 38:119147. doi: 10.1016/j.gep.2020.119147 32987154 PMC7726062

[10] Wu H Zhu X Zhou H Sha M Ye J Yu H. Mitochondrial ribosomal proteins and cancer. Medicina (Kaunas). (2025) 61:96. doi: 10.3390/medicina61010096 39859078 PMC11766452

[11] Greber BJ Bieri P Leibundgut M Leitner A Aebersold R Boehringer D. Ribosome. The complete structure of the 55S mammalian mitochondrial ribosome. Science. (2015) 348:303–8. doi: 10.1126/science.aaa3872 25837512

[12] Lv M Zhou W Hao Y Li F Zhang H Yao X. Structural insights into the specific recognition of mitochondrial ribosome-binding factor hsRBFA and 12 S rRNA by methyltransferase METTL15. Cell Discovery. (2024) 10:11. doi: 10.1038/s41421-023-00634-z 38291322 PMC10828496

[13] Sharma MR Koc EC Datta PP Booth TM Spremulli LL Agrawal RK. Structure of the mammalian mitochondrial ribosome reveals an expanded functional role for its component proteins. Cell. (2003) 115:97–108. doi: 10.1016/s0092-8674(03)00762-1 14532006

[14] Kaushal PS Sharma MR Agrawal RK. The 55S mammalian mitochondrial ribosome and its tRNA-exit region. Biochimie. (2015) 114:119–26. doi: 10.1016/j.biochi.2015.03.013 PMC4772884 25797916

[15] Koripella RK Deep A Agrawal EK Keshavan P Banavali NK Agrawal RK. Distinct mechanisms of the human mitoribosome recycling and antibiotic resistance. Nat Commun. (2021) 12:3607. doi: 10.1038/s41467-021-23726-4 34127662 PMC8203779

[16] Amunts A Brown A Toots J Scheres SHW Ramakrishnan V. Ribosome. The structure of the human mitochondrial ribosome. Science. (2015) 348:95–8. doi: 10.1126/science.aaa1193 PMC4501431 25838379

[17] Pham TCP Raun SH Havula E Henriquez-Olguín C Rubalcava-Gracia D Frank E. The mitochondrial mRNA-stabilizing protein SLIRP regulates skeletal muscle mitochondrial structure and respiration by exercise-recoverable mechanisms. Nat Commun. (2024) 15:9826. doi: 10.1038/s41467-024-54183-4 39537626 PMC11561311

[18] Li X Wang M Li S Chen Y Wang M Wu Z. HIF-1-induced mitochondrial ribosome protein L52: a mechanism for breast cancer cellular adaptation and metastatic initiation in response to hypoxia. Theranostics. (2021) 11:7337–59. doi: 10.7150/thno.57804 PMC8210597 34158854

[19] Wang Z Li J Long X Jiao L Zhou M Wu K. MRPS16 facilitates tumor progression via the PI3K/AKT/Snail signaling axis. J Cancer. (2020) 11:2032–43. doi: 10.7150/jca.39671 PMC7052926 32127931

[20] Rai AK Sanghvi S Muthukumaran NS Chandrasekera D Kadam A Kishore J. Role of mitochondrial ribosomal protein L7/L12 (MRPL12) in diabetic ischemic heart disease. Free Radic Biol Med. (2024) 222:531–8. doi: 10.1016/j.freeradbiomed.2024.07.003 PMC12935059 38977138

[21] Serre V Rozanska A Beinat M Chretien D Boddaert N Munnich A. Mutations in mitochondrial ribosomal protein MRPL12 leads to growth retardation, neurological deterioration and mitochondrial translation deficiency. Biochim Biophys Acta. (2013) 1832:1304–12. doi: 10.1016/j.bbadis.2013.04.014 PMC3787750 23603806

[22] Yang J Shay C Saba NF Teng Y. Cancer metabolism and carcinogenesis. Exp Hematol Oncol. (2024) 13:10. doi: 10.1186/s40164-024-00482-x 38287402 PMC10826200

[23] Zheng J. Energy metabolism of cancer: Glycolysis versus oxidative phosphorylation (Review). Oncol Lett. (2012) 4:1151–7. doi: 10.3892/ol.2012.928 PMC3506713 23226794

[24] Denko NC. Hypoxia, HIF1 and glucose metabolism in the solid tumour. Nat Rev Cancer. (2008) 8:705–13. doi: 10.1038/nrc2468 19143055

[25] Lee YK Lim JJ Jeoun UW Min S Lee EB Kwon SM. Lactate-mediated mitoribosomal defects impair mitochondrial oxidative phosphorylation and promote hepatoma cell invasiveness. J Biol Chem. (2017) 292:20208–17. doi: 10.1074/jbc.M117.809012 PMC5724007 28978646

[26] Song BS Moon JS Tian J Lee HY Sim BC Kim SH. Mitoribosomal defects aggravate liver cancer via aberrant glycolytic flux and T cell exhaustion. J Immunother Cancer. (2022) 10:e004337. doi: 10.1136/jitc-2021-004337 35580931 PMC9114962

[27] Tufail M Jiang CH Li N. Altered metabolism in cancer: insights into energy pathways and therapeutic targets. Mol Cancer. (2024) 23:203. doi: 10.1186/s12943-024-02119-3 39294640 PMC11409553

[28] Łukasiewicz S Czeczelewski M Forma A Baj J Sitarz R Stanisławek A. Breast cancer-epidemiology, risk factors, classification, prognostic markers, and current treatment strategies-an updated review. Cancers (Basel). (2021) 13:4287. doi: 10.3390/cancers13174287 34503097 PMC8428369

[29] Xu YH Deng JL Wang LP Zhang HB Tang L Huang Y. Identification of candidate genes associated with breast cancer prognosis. DNA Cell Biol. (2020) 39:1205–27. doi: 10.1089/dna.2020.5482 32456464

[30] Ye H Zhang N. Identification of the upregulation of MRPL13 as a novel prognostic marker associated with overall survival time and immunotherapy response in breast cancer. Comput Math Methods Med. (2021) 2021:1498924. doi: 10.1155/2021/1498924 34868337 PMC8639240

[31] Yin J Lin C Jiang M Tang X Xie D Chen J. CENPL, ISG20L2, LSM4, MRPL3 are four novel hub genes and may serve as diagnostic and prognostic markers in breast cancer. Sci Rep. (2021) 11:15610. doi: 10.1038/s41598-021-95068-6 34341433 PMC8328991

[32] Liu Y Sun H Li X Liu Q Zhao Y Li L. Identification of a three-RNA binding proteins (RBPs) signature predicting prognosis for breast cancer. Front Oncol. (2021) 11:663556. doi: 10.3389/fonc.2021.663556 34322380 PMC8311660

[33] Lin X Guo L Lin X Wang Y Zhang G. Expression and prognosis analysis of mitochondrial ribosomal protein family in breast cancer. Sci Rep. (2022) 12:10658. doi: 10.1038/s41598-022-14724-7 35739158 PMC9226049

[34] Buchynska LG Iurchenko NP Kashuba EV Brieieva OV Glushchenko NM Mints M. Overexpression of the mitochondrial ribosomal protein S18–2 in the invasive breast carcinomas. Exp Oncol. (2018) 40:303–8. doi: 10.31768/2312-8852.2018.40(4):303-308 30593750

[35] Oviya RP Thangaretnam KP Ramachandran B Ramanathan P Jayavelu S Gopal G. Mitochondrial ribosomal small subunit (MRPS) MRPS23 protein-protein interaction reveals phosphorylation by CDK11-p58 affecting cell proliferation and knockdown of MRPS23 sensitizes breast cancer cells to CDK1 inhibitors. Mol Biol Rep. (2022) 49:9521–34. doi: 10.1007/s11033-022-07842-y 35962848

[36] Quigley DA Fiorito E Nord S Van Loo P Alnæs GG Fleischer T. The 5p12 breast cancer susceptibility locus affects MRPS30 expression in estrogen-receptor positive tumors. Mol Oncol. (2014) 8:273–84. doi: 10.1016/j.molonc.2013.11.008 PMC3964809 24388359

[37] Guo X Lin W Bao J Cai Q Pan X Bai M. A comprehensive cis-eQTL analysis revealed target genes in breast cancer susceptibility loci identified in genome-wide association studies. Am J Hum Genet. (2018) 102:890–903. doi: 10.1016/j.ajhg.2018.03.016 29727689 PMC5986971

[38] Wu B Pan Y Liu G Yang T Jin Y Zhou F. MRPS30-DT knockdown inhibits breast cancer progression by targeting Jab1/Cops5. Front Oncol. (2019) 9:1170. doi: 10.3389/fonc.2019.01170 31788446 PMC6854119

[39] Ghoussaini M French JD Michailidou K Nord S Beesley J Canisus S. Evidence that the 5p12 Variant rs10941679 Confers Susceptibility to Estrogen-Receptor-Positive Breast Cancer through FGF10 and MRPS30 Regulation. Am J Hum Genet. (2016) 99:903–11. doi: 10.1016/j.ajhg.2016.07.017 PMC5065698 27640304

[40] Bhatti P Doody MM Rajaraman P Alexander BH Yeager M Hutchinson A. Novel breast cancer risk alleles and interaction with ionizing radiation among U.S. radiologic technologists. Radiat Res. (2010) 173:214–24. doi: 10.1667/RR1985.1 PMC2922870 20095854

[41] Zhang Y Manjunath M Zhang S Chasman D Roy S Song JS. Integrative genomic analysis predicts causative Cis-regulatory mechanisms of the breast cancer-associated genetic variant rs4415084. Cancer Res. (2018) 78:1579–91. doi: 10.1158/0008-5472.CAN-17-3486 PMC5882544 29351903

[42] Wang K Li L Fu L Yuan Y Dai H Zhu T. Integrated bioinformatics analysis the function of RNA binding proteins (RBPs) and their prognostic value in breast cancer. Front Pharmacol. (2019) 10:140. doi: 10.3389/fphar.2019.00140 30881302 PMC6405693

[43] Tao Z Suo H Zhang L Jin Z Wang Z Wang D. MRPL13 is a prognostic cancer biomarker and correlates with immune infiltrates in breast cancer. Onco Targets Ther. (2020) 13:12255–68. doi: 10.2147/OTT.S263998 PMC7708783 33273831

[44] Zhou X Xiao C Han T Qiu S Wang M Chu J. Prognostic biomarkers related to breast cancer recurrence identified based on Logit model analysis. World J Surg Oncol. (2020) 18:254. doi: 10.1186/s12957-020-02026-z 32977823 PMC7519567

[45] Cai M Li H Chen R Zhou X. MRPL13 promotes tumor cell proliferation, migration and EMT process in breast cancer through the PI3K-AKT-mTOR pathway. Cancer Manag Res. (2021) 13:2009–24. doi: 10.2147/CMAR.S296038 PMC7920513 33658859

[46] Zheng C Yao H Lu L Li H Zhou L He X. Dysregulated ribosome biogenesis is a targetable vulnerability in triple-negative breast cancer: MRPS27 as a key mediator of the stemness-inhibitory effect of lovastatin. Int J Biol Sci. (2024) 20(6):2130–48. doi: 10.7150/ijbs.94058 PMC11008279 38617541

[47] Klæstad E Opdahl S Engstrøm MJ Ytterhus B Wik E Bofin AM. MRPS23 amplification and gene expression in breast cancer; association with proliferation and the non-basal subtypes. Breast Cancer Res Treat. (2020) 180:73–86. doi: 10.1007/s10549-020-05532-6 31950385 PMC7031208

[48] Gao Y Li F Zhou H Yang Y Wu R Chen Y. Down-regulation of MRPS23 inhibits rat breast cancer proliferation and metastasis. Oncotarget. (2017) 8:71772–81. doi: 10.18632/oncotarget.17888 PMC5641088 29069745

[49] Oviya RP Gopal G Shirley SS Sridevi V Jayavelu S Rajkumar T. Mitochondrial ribosomal small subunit proteins (MRPS) MRPS6 and MRPS23 show dysregulation in breast cancer affecting tumorigenic cellular processes. Gene. (2021) 790:145697. doi: 10.1016/j.gene.2021.145697 33964376

[50] Li Y Wu X Yang P Jiang G Luo Y. Machine learning for lung cancer diagnosis, treatment, and prognosis. Genomics Proteomics Bioinf. (2022) 20:850–66. doi: 10.1016/j.gpb.2022.11.003 PMC10025752 36462630

[51] Ren F Fei Q Qiu K Zhang Y Zhang H Sun L. Liquid biopsy techniques and lung cancer: diagnosis, monitoring and evaluation. J Exp Clin Cancer Res. (2024) 43:96. doi: 10.1186/s13046-024-03026-7 38561776 PMC10985944

[52] Zhong X He Z Fan Y Yin L Hong Z Tong Y. Multi-omics analysis of MRPL-13 as a tumor-promoting marker from pan-cancer to lung adenocarcinoma. Aging (Albany NY). (2023) 15:10640–80. doi: 10.18632/aging.205104 PMC10599762 37827692

[53] Jing C Fu R Wang C Li X Zhang W. MRPL13 act as a novel therapeutic target and could promote cell proliferation in non-small cell lung cancer. Cancer Manag Res. (2021) 13:5535–45. doi: 10.2147/CMAR.S316428 PMC8285246 34285575

[54] Sun Y Liu Y Wang P Chang L Huang J. The m6A reader YTHDC2 suppresses lung adenocarcinoma tumorigenesis by destabilizing MRPL12. Mol Biotechnol. (2024) 66:1051–61. doi: 10.1007/s12033-023-01002-8 38129673

[55] Ji X Zhang T Sun J Song X Ma G Xu L. UBASH3B-mediated MRPL12 Y60 dephosphorylation inhibits LUAD development by driving mitochondrial metabolism reprogramming. J Exp Clin Cancer Res. (2024) 43:268. doi: 10.1186/s13046-024-03181-x 39343960 PMC11441236

[56] Chengcheng L Raza SHA Shengchen Y Mohammedsaleh ZM Shater AF Saleh FM. Bioinformatics role of the WGCNA analysis and co-expression network identifies of prognostic marker in lung cancer. Saudi J Biol Sci. (2022) 29:3519–27. doi: 10.1016/j.sjbs.2022.02.016 PMC9280221 35844396

[57] Hu Y Liu Y Ma C Ai K. MRPL12 acts as A novel prognostic biomarker involved in immune cell infiltration and tumor progression of lung adenocarcinoma. Int J Mol Sci. (2023) 24:2762. doi: 10.3390/ijms24032762 36769082 PMC9917664

[58] Jiang W Zhang C Kang Y Yu X Pang P Li G. MRPL42 is activated by YY1 to promote lung adenocarcinoma progression. J Cancer. (2021) 12:2403–11. doi: 10.7150/jca.52277 PMC7974901 33758616

[59] Wei D Sun D Sirera R Afzal MZ Leong TL Li X. Overexpression of MRPL19 in predicting poor prognosis and promoting the development of lung adenocarcinoma. Transl Lung Cancer Res. (2023) 12:1517–38. doi: 10.21037/tlcr-23-306 PMC10413030 37577299

[60] Zeng Y Shi Y Xu L Zeng Y Cui X Wang Y. Prognostic value and related regulatory networks of MRPL15 in non-small-cell lung cancer. Front Oncol. (2021) 11:656172. doi: 10.3389/fonc.2021.656172 34026630 PMC8138120

[61] Li XY He XY Zhao H Qi L Lu JJ. Identification of a novel therapeutic target for lung cancer: Mitochondrial ribosome protein L9. Pathol Res Pract. (2023) 248:154625. doi: 10.1016/j.prp.2023.154625 37343379

[62] Yoo YA Kim MJ Park JK Chung YM Lee JH Chi SG. Mitochondrial ribosomal protein L41 suppresses cell growth in association with p53 and p27Kip1. Mol Cell Biol. (2005) 25:6603–16. doi: 10.1128/MCB.25.15.6603-6616.2005 PMC1190350 16024796

[63] Sung H Ferlay J Siegel RL Laversanne M Soerjomataram I Jemal A. Global cancer statistics 2020: GLOBOCAN estimates of incidence and mortality worldwide for 36 cancers in 185 countries. CA Cancer J Clin. (2021) 71:209–49. doi: 10.3322/caac.21660 33538338

[64] Zhang L Lu P Yan L Yang L Wang Y Chen J. MRPL35 is up-regulated in colorectal cancer and regulates colorectal cancer cell growth and apoptosis. Am J Pathol. (2019) 189:1105–20. doi: 10.1016/j.ajpath.2019.02.003 30862482

[65] Yan HX Zhang YJ Zhang Y Ren X Shen YF Cheng MB. CRIF1 enhances p53 activity via the chromatin remodeler SNF5 in the HCT116 colon cancer cell lines. Biochim Biophys Acta Gene Regul Mech. (2017) 1860:516–22. doi: 10.1016/j.bbagrm.2017.02.006 28235567

[66] Zhu X Liu Y Xu J Cheng Z Yu Y Chu M. miR-608 rs4919510 polymorphism may affect susceptibility to colorectal cancer by upregulating MRPL43 expression. DNA Cell Biol. (2020) 39:2017–27. doi: 10.1089/dna.2020.5689 33147064

[67] Liu L Luo C Luo Y Chen L Liu Y Wang Y. MRPL33 and its splicing regulator hnRNPK are required for mitochondria function and implicated in tumor progression. Oncogene. (2018) 37:86–94. doi: 10.1038/onc.2017.314 28869607

[68] Li P Hao Z Liu H Zhu B Dang L Ma C. Quantitative proteomics analysis of berberine-treated colon cancer cells reveals potential therapy targets. Biol (Basel). (2021) 10:250. doi: 10.3390/biology10030250 PMC8005188 33806918

[69] Abdul Aziz NA Mokhtar NM Harun R Mollah MM Mohamed Rose I Sagap I. A 19-Gene expression signature as a predictor of survival in colorectal cancer. BMC Med Genomics. (2016) 9:58. doi: 10.1186/s12920-016-0218-1 27609023 PMC5016995

[70] Sui L Zeng J Zhao H Ye L Martin TA Sanders AJ. Death associated protein−3 (DAP3) and DAP3 binding cell death enhancer−1 (DELE1) in human colorectal cancer, and their impacts on clinical outcome and chemoresistance. Int J Oncol. (2023) 62:7. doi: 10.3892/ijo.2022.5455 36382667 PMC9728556

[71] Luo D Liu Y Lu Z Huang L. Targeted therapy and immunotherapy for gastric cancer: rational strategies, novel advancements, challenges, and future perspectives. Mol Med. (2025) 31:52. doi: 10.1186/s10020-025-01075-y 39923010 PMC11806620

[72] Yuan L Li JX Yang Y Chen Y Ma TT Liang S. Depletion of MRPL35 inhibits gastric carcinoma cell proliferation by regulating downstream signaling proteins. World J Gastroenterol. (2021) 27:1785–804. doi: 10.3748/wjg.v27.i16.1785 PMC8072187 33967557

[73] Yuan L Yang Y Li X Zhou X Du YH Liu WJ. 18β-glycyrrhetinic acid regulates mitochondrial ribosomal protein L35-associated apoptosis signaling pathways to inhibit proliferation of gastric carcinoma cells. World J Gastroenterol. (2022) 28:2437–56. doi: 10.3748/wjg.v28.i22.2437 PMC9258276 35979263

[74] Zhou W Ouyang J Li J Liu F An T Cheng L. MRPS17 promotes invasion and metastasis through PI3K/AKT signal pathway and could be potential prognostic marker for gastric cancer. J Cancer. (2021) 12:4849–61. doi: 10.7150/jca.55719 PMC8247386 34234855

[75] Li J Zhou W Wei J Xiao X An T Wu W. Prognostic value and biological functions of RNA binding proteins in stomach adenocarcinoma. Onco Targets Ther. (2021) 14:1689–705. doi: 10.2147/OTT.S297973 PMC7942957 33707953

[76] Yu MJ Zhao N Shen H Wang H. Long noncoding RNA MRPL39 inhibits gastric cancer proliferation and progression by directly targeting miR-130. Genet Test Mol Biomarkers. (2018) 22:656–63. doi: 10.1089/gtmb.2018.0151 30452299

[77] Zhou L Wu Y Xin L Zhou Q Li S Yuan Y. Development of RNA binding proteins expression signature for prognosis prediction in gastric cancer patients. Am J Transl Res. (2020) 12:6775–92. PMC7653620 33194072

[78] Jia Y Ye L Ji K Zhang L Hargest R Ji J. Death-associated protein-3, DAP-3, correlates with preoperative chemotherapy effectiveness and prognosis of gastric cancer patients following perioperative chemotherapy and radical gastrectomy. Br J Cancer. (2014) 110:421–9. doi: 10.1038/bjc.2013.712 PMC3899757 24300973

[79] Ye J Lin Y Liao Z Gao X Lu C Lu L. Single cell-spatial transcriptomics and bulk multi-omics analysis of heterogeneity and ecosystems in hepatocellular carcinoma. NPJ Precis Oncol. (2024) 8:262. doi: 10.1038/s41698-024-00752-1 39548284 PMC11568154

[80] Yuan Y Ju YS Kim Y Li J Wang Y Yoon CJ. Comprehensive molecular characterization of mitochondrial genomes in human cancers. Nat Genet. (2020) 52:342–52. doi: 10.1038/s41588-019-0557-x PMC7058535 32024997

[81] Zhou C Chen Z Peng C Chen C Li H. Long noncoding RNA TRIM52-AS1 sponges miR-514a-5p to facilitate hepatocellular carcinoma progression through increasing MRPS18A. Cancer Biother Radiopharm. (2021) 36:211–9. doi: 10.1089/cbr.2019.3271 32391716

[82] Pu M Wang J Huang Q Zhao G Xia C Shang R. High MRPS23 expression contributes to hepatocellular carcinoma proliferation and indicates poor survival outcomes. Tumour Biol. (2017) 39:1010428317709127. doi: 10.1177/1010428317709127 28714366

[83] Ye J Li H Wei J Luo Y Liu H Zhang J. Risk Scoring System based on lncRNA Expression for Predicting Survival in Hepatocellular Carcinoma with Cirrhosis. Asian Pac J Cancer Prev. (2020) 21:1787–95. doi: 10.31557/APJCP.2020.21.6.1787 PMC7568908 32592379

[84] Wei Z Jia J Heng G Xu H Shan J Wang G. Sirtuin-1/mitochondrial ribosomal protein S5 axis enhances the metabolic flexibility of liver cancer stem cells. Hepatology. (2019) 70:1197–213. doi: 10.1002/hep.30622 30901096

[85] Lin YX Pan JY Feng WD Huang TC Li CZ. MRPL48 is a novel prognostic and predictive biomarker of hepatocellular carcinoma. Eur J Med Res. (2023) 28:589. doi: 10.1186/s40001-023-01571-z 38093387 PMC10720175

[86] Min S Lee YK Hong J Park TJ Woo HG Kwon SM. MRPS31 loss is a key driver of mitochondrial deregulation and hepatocellular carcinoma aggressiveness. Cell Death Dis. (2021) 12:1076. doi: 10.1038/s41419-021-04370-8 34772924 PMC8589861

[87] Sultana N Rahman M Myti S Islam J Mustafa MG Nag K. A novel knowledge-derived data potentizing method revealed unique liver cancer-associated genetic variants. Hum Genomics. (2019) 13:30. doi: 10.1186/s40246-019-0213-7 31272500 PMC6610914

[88] Liu J Lu J Li W. A comprehensive prognostic and immunological analysis of a new three-gene signature in hepatocellular carcinoma. Stem Cells Int. (2021) 2021:5546032. doi: 10.1155/2021/5546032 34188686 PMC8192212

[89] Xie C Hu J Hu Q Jiang L Chen W. Classification of the mitochondrial ribosomal protein-associated molecular subtypes and identified a serological diagnostic biomarker in hepatocellular carcinoma. Front Surg. (2023) 9:1062659. doi: 10.3389/fsurg.2022.1062659 36684217 PMC9853988

[90] Tang B Zhu J Zhao Z Lu C Liu S Fang S. Diagnosis and prognosis models for hepatocellular carcinoma patient’s management based on tumor mutation burden. J Adv Res. (2021) 33:153–65. doi: 10.1016/j.jare.2021.01.018 PMC8463909 34603786

[91] Li M Liu Z Wang J Liu H Gong H Li S. Systematic analysis identifies a specific RNA-binding protein-related gene model for prognostication and risk-adjustment in HBV-related hepatocellular carcinoma. Front Genet. (2021) 12:707305. doi: 10.3389/fgene.2021.707305 34422009 PMC8371711

[92] Ji X Yang Z Li C Zhu S Zhang Y Xue F. Mitochondrial ribosomal protein L12 potentiates hepatocellular carcinoma by regulating mitochondrial biogenesis and metabolic reprogramming. Metabolism. (2024) 152:155761. doi: 10.1016/j.metabol.2023.155761 38104924

[93] Argiris A Karamouzis MV Raben D Ferris RL. Head and neck cancer. Lancet. (2008) 371:1695–709. doi: 10.1016/S0140-6736(08)60728-X PMC7720415 18486742

[94] Koc EC Haciosmanoglu E Claudio PP Wolf A Califano L Friscia M. Impaired mitochondrial protein synthesis in head and neck squamous cell carcinoma. Mitochondrion. (2015) 24:113–21. doi: 10.1016/j.mito.2015.07.123 26238294

[95] Zhang HM Li ZY Dai ZT Wang J Li LW Zong QB. Interaction of MRPL9 and GGCT promotes cell proliferation and migration by activating the MAPK/ERK pathway in papillary thyroid cancer. Int J Mol Sci. (2022) 23:11989. doi: 10.3390/ijms231911989 36233293 PMC9570013

[96] Kim HJ Nguyen QK Jung SN Lim MA Oh C Piao Y. Mitochondrial ribosomal protein L14 promotes cell growth and invasion by modulating reactive oxygen species in thyroid cancer. Clin Exp Otorhinolaryngol. (2023) 16:184–97. doi: 10.21053/ceo.2022.01760 PMC10208851 36822197

[97] Lee J Seol MY Jeong S Lee CR Ku CR Kang SW. A metabolic phenotype based on mitochondrial ribosomal protein expression as a predictor of lymph node metastasis in papillary thyroid carcinoma. Med (Baltimore). (2015) 94:e380. doi: 10.1097/MD.0000000000000380 PMC4602546 25590838

[98] Chen CW Fu M Du ZH Zhao F Yang WW Xu LH. Long noncoding RNA MRPL23-AS1 promotes adenoid cystic carcinoma lung metastasis. Cancer Res. (2020) 80:2273–85. doi: 10.1158/0008-5472.CAN-19-0819 32098781

[99] Yuan Z Yu Y Zhang B Miao L Wang L Zhao K. Genetic variants in lncRNA H19 are associated with the risk of oral squamous cell carcinoma in a Chinese population. Oncotarget. (2018) 9:23915–22. doi: 10.18632/oncotarget.23673 PMC5963630 29844862

[100] Zou X Hu X He F Zhang M Kong X Rui S. LncRNA LINC00152 promotes oral squamous cell carcinoma growth via enhancing Upstream Transcription Factor 1 mediated Mitochondrial Ribosomal Protein L52 transcription. J Oral Pathol Med. (2022) 51:454–63. doi: 10.1111/jop.13253 34664331

[101] Guo T Zambo KDA Zamuner FT Ou T Hopkins C Kelley DZ. Chromatin structure regulates cancer-specific alternative splicing events in primary HPV-related oropharyngeal squamous cell carcinoma. Epigenetics. (2020) 15:959–71. doi: 10.1080/15592294.2020.1741757 PMC7518675 32164487

[102] Hu G Jiang Q Liu L Peng H Wang Y Li S. Integrated analysis of RNA-binding proteins associated with the prognosis and immunosuppression in squamous cell carcinoma of head and neck. Front Genet. (2021) 11:571403. doi: 10.3389/fgene.2020.571403 33505420 PMC7831273

[103] Al Ageeli E. Alterations of mitochondria and related metabolic pathways in leukemia: A narrative review. Saudi J Med Med Sci. (2020) 8:3–11. doi: 10.4103/sjmms.sjmms_112_18 31929772 PMC6945320

[104] Bao S Wang X Li M Gao Z Zheng D Shen D. Potential of mitochondrial ribosomal genes as cancer biomarkers demonstrated by bioinformatics results. Front Oncol. (2022) 12:835549. doi: 10.3389/fonc.2022.835549 35719986 PMC9204274

[105] Ran Q Hao P Xiao Y Xiang L Ye X Deng X. CRIF1 interacting with CDK2 regulates bone marrow microenvironment-induced G0/G1 arrest of leukemia cells. PloS One. (2014) 9:e85328. doi: 10.1371/journal.pone.0085328 24520316 PMC3919709

[106] Vahedi S Chueh FY Dutta S Chandran B Yu CL. Nuclear lymphocyte-specific protein tyrosine kinase and its interaction with CR6-interacting factor 1 promote the survival of human leukemic T cells. Oncol Rep. (2015) 34:43–50. doi: 10.3892/or.2015.3990 25997448 PMC4484609

[107] Gao HY Wang W Luo XG Jiang YF He X Xu P. Screening of prognostic risk microRNAs for acute myeloid leukemia. Hematology. (2018) 23:747–55. doi: 10.1080/10245332.2018.1475860 29781401

[108] Lebedev TD Vagapova ER Popenko VI Leonova OG Spirin PV Prassolov VS. Two receptors, two isoforms, two cancers: comprehensive analysis of KIT and trkA expression in neuroblastoma and acute myeloid leukemia. Front Oncol. (2019) 9:1046. doi: 10.3389/fonc.2019.01046 31681584 PMC6813278

[109] Lan Z Li X Zhang X. Glioblastoma: an update in pathology, molecular mechanisms and biomarkers. Int J Mol Sci. (2024) 25:3040. doi: 10.3390/ijms25053040 38474286 PMC10931698

[110] Louis DN Perry A Wesseling P Brat DJ Cree IA Figarella-Branger D. The 2021 WHO classification of tumors of the central nervous system: a summary. Neuro Oncol. (2021) 23:1231–51. doi: 10.1093/neuonc/noab106 PMC8328013 34185076

[111] Agnihotri S Zadeh G. Metabolic reprogramming in glioblastoma: the influence of cancer metabolism on epigenetics and unanswered questions. Neuro Oncol. (2016) 18:160–72. doi: 10.1093/neuonc/nov125 PMC4724176 26180081

[112] Chen L Zhang H Shang C Hong Y. The role and applied value of mitochondria in glioma-related research. CNS Neurosci Ther. (2024) 30:e70121. doi: 10.1111/cns.70121 39639571 PMC11621238

[113] Hao C Duan H Li H Wang H Liu Y Fan Y. Knockdown of MRPL42 suppresses glioma cell proliferation by inducing cell cycle arrest and apoptosis. Biosci Rep. (2018) 38:BSR20171456. doi: 10.1042/BSR20171456 29531015 PMC5920136

[114] Alshabi AM Vastrad B Shaikh IA Vastrad C. Identification of crucial candidate genes and pathways in glioblastoma multiform by bioinformatics analysis. Biomolecules. (2019) 9:201. doi: 10.3390/biom9050201 31137733 PMC6571969

[115] Weiderpass E Labrèche F. Malignant tumors of the female reproductive system. Saf Health Work. (2012) 3:166–80. doi: 10.5491/SHAW.2012.3.3.166 PMC3443692 23019529

[116] Xu H Zou R Li F Liu J Luan N Wang S. MRPL15 is a novel prognostic biomarker and therapeutic target for epithelial ovarian cancer. Cancer Med. (2021) 10:3655–73. doi: 10.1002/cam4.3907 PMC8178508 33934540

[117] Qiu X Guo D Du J Bai Y Wang F. A novel biomarker, MRPS12 functions as a potential oncogene in ovarian cancer and is a promising prognostic candidate. Med (Baltimore). (2021) 100:e24898. doi: 10.1097/MD.0000000000024898 PMC7909224 33663122

[118] Mints M Mushtaq M Iurchenko N Kovalevska L Stip MC Budnikova D. Mitochondrial ribosomal protein S18–2 is highly expressed in endometrial cancers along with free E2F1. Oncotarget. (2016) 7:22150–8. doi: 10.18632/oncotarget.7905 PMC5008351 26959119

[119] Lyng H Brøvig RS Svendsrud DH Holm R Kaalhus O Knutstad K. Gene expressions and copy numbers associated with metastatic phenotypes of uterine cervical cancer. BMC Genomics. (2006) 7:268. doi: 10.1186/1471-2164-7-268 17054779 PMC1626467

[120] Chung HK Yi YW Jung NC Kim D Suh JM Kim H. CR6-interacting factor 1 interacts with Gadd45 family proteins and modulates the cell cycle. J Biol Chem. (2003) 278:28079–88. doi: 10.1074/jbc.M212835200 12716909

[121] Jang HN Moon SJ Jung KC Kim SW Kim H Han D. Mass spectrometry-based proteomic discovery of prognostic biomarkers in adrenal cortical carcinoma. Cancers (Basel). (2021) 13:3890. doi: 10.3390/cancers13153890 34359790 PMC8345732

[122] Mushtaq M Jensen L Davidsson S Grygoruk OV Andrén O Kashuba V. The MRPS18–2 protein levels correlate with prostate tumor progression and it induces CXCR4-dependent migration of cancer cells. Sci Rep. (2018) 8:2268. doi: 10.1038/s41598-018-20765-8 29396484 PMC5797078

[123] Chen Y Cairns R Papandreou I Koong A Denko NC. Oxygen consumption can regulate the growth of tumors, a new perspective on the Warburg effect. PloS One. (2009) 4:e7033. doi: 10.1371/journal.pone.0007033 19753307 PMC2737639

[124] Wu Y Zhang L He S Guan B He A Yang K. Identification of immune-related LncRNA for predicting prognosis and immunotherapeutic response in bladder cancer. Aging (Albany NY). (2020) 12:23306–25. doi: 10.18632/aging.104115 PMC7746369 33221763

[125] Hepburn AC Lazzarini N Veeratterapillay R Wilson L Bacardit J Heer R. Identification of CNGB1 as a predictor of response to neoadjuvant chemotherapy in muscle-invasive bladder cancer. Cancers (Basel). (2021) 13:3903. doi: 10.3390/cancers13153903 34359804 PMC8345622

[126] Liu J Wu S Xie X Wang Z Lei Q. Identification of potential crucial genes and key pathways in osteosarcoma. Hereditas. (2020) 157:29. doi: 10.1186/s41065-020-00142-0 32665038 PMC7362476

[127] Decock A Ongenaert M Cannoodt R Verniers K De Wilde B Laureys G. Methyl-CpG-binding domain sequencing reveals a prognostic methylation signature in neuroblastoma. Oncotarget. (2016) 7:1960–72. doi: 10.18632/oncotarget.6477 PMC4811509 26646589

[128] Tian A Pu K Li B Li M Liu X Gao L. Weighted gene coexpression network analysis reveals hub genes involved in cholangiocarcinoma progression and prognosis. Hepatol Res. (2019) 49:1195–206. doi: 10.1111/hepr.13386 PMC6899837 31177590

[129] Zhuang L Meng Z Yang Z. MRPL27 contributes to unfavorable overall survival and disease-free survival from cholangiocarcinoma patients. Int J Med Sci. (2021) 18:936–43. doi: 10.7150/ijms.50782 PMC7807179 33456351

[130] Zhao DD Zhao X Li WT. Identification of differentially expressed metastatic genes and their signatures to predict the overall survival of uveal melanoma patients by bioinformatics analysis. Int J Ophthalmol. (2020) 13:1046–53. doi: 10.18240/ijo.2020.07.05 PMC7321956 32685390

[131] de Visser KE Joyce JA. The evolving tumor microenvironment: From cancer initiation to metastatic outgrowth. Cancer Cell. (2023) 41:374–403. doi: 10.1016/j.ccell.2023.02.016 36917948

[132] Anderson NM Simon MC. The tumor microenvironment. Curr Biol. (2020) 30:R921–5. doi: 10.1016/j.cub.2020.06.081 PMC8194051 32810447

[133] Quail DF Joyce JA. Microenvironmental regulation of tumor progression and metastasis. Nat Med. (2013) 19:1423–37. doi: 10.1038/nm.3394 PMC3954707 24202395

[134] Behera BP Mishra SR Patra S Mahapatra KK Bhol CS Panigrahi DP. Molecular regulation of mitophagy signaling in tumor microenvironment and its targeting for cancer therapy. Cytokine Growth Factor Rev. (2025), S1359–6101(25)00004-8. doi: 10.1016/j.cytogfr.2025.01.004 PMC12267565 39880721

[135] Angajala A Lim S Phillips JB Kim JH Yates C You Z. Diverse roles of mitochondria in immune responses: novel insights into immuno-metabolism. Front Immunol. (2018) 9:1605. doi: 10.3389/fimmu.2018.01605 30050539 PMC6052888

[136] Liu L Shao M Huang Y Qian P Huang H. Unraveling the roles and mechanisms of mitochondrial translation in normal and Malignant hematopoiesis. J Hematol Oncol. (2024) 17:95. doi: 10.1186/s13045-024-01615-9 39396039 PMC11470598

[137] Xie XQ Yang Y Wang Q Liu HF Fang XY Li CL. Targeting ATAD3A-PINK1-mitophagy axis overcomes chemoimmunotherapy resistance by redirecting PD-L1 to mitochondria. Cell Res. (2023) 33:215–28. doi: 10.1038/s41422-022-00766-z PMC9977947 36627348

[138] Kwon SM Lee YK Min S Woo HG Wang HJ Yoon G. Mitoribosome defect in hepatocellular carcinoma promotes an aggressive phenotype with suppressed immune reaction. iScience. (2020) 23:101247. doi: 10.1016/j.isci.2020.101247 32629612 PMC7306587

[139] Wong KKL Verheyen EM. Metabolic reprogramming in cancer: mechanistic insights from Drosophila. Dis Model Mech. (2021) 14:1–17. doi: 10.1242/dmm.048934 34240146 PMC8277969

[140] Li X Wang M Denk T Buschauer R Li Y Beckmann R. Structural basis for differential inhibition of eukaryotic ribosomes by tigecycline. Nat Commun. (2024) 15:5481. doi: 10.1038/s41467-024-49797-7 38942792 PMC11213857

[141] van den Bogert C Dontje BH Holtrop M Melis TE Romijn JC van Dongen JW. Arrest of the proliferation of renal and prostate carcinomas of human origin by inhibition of mitochondrial protein synthesis. Cancer Res. (1986) 46:3283–9. 3011245

[142] Yang B Lu Y Zhang A Zhou A Zhang L Zhang L. Doxycycline induces apoptosis and inhibits proliferation and invasion of human cervical carcinoma stem cells. PloS One. (2015) 10:e0129138. doi: 10.1371/journal.pone.0129138 26111245 PMC4482382

[143] Kim HJ Maiti P Barrientos A. Mitochondrial ribosomes in cancer. Semin Cancer Biol. (2017) 47:67–81. doi: 10.1016/j.semcancer.2017.04.004 28445780 PMC5662495

[144] Pontieri P Hartings H Di Salvo M Massardo DR De Stefano M Pizzolante G. Mitochondrial ribosomal proteins involved in tellurite resistance in yeast Saccharomyces cerevisiae. Sci Rep. (2018) 8:12022. doi: 10.1038/s41598-018-30479-6 30104660 PMC6089990

[145] Sotgia F Fiorillo M Lisanti MP. Mitochondrial markers predict recurrence, metastasis and tamoxifen-resistance in breast cancer patients: Early detection of treatment failure with companion diagnostics. Oncotarget. (2017) 8:68730–45. doi: 10.18632/oncotarget.19612 PMC5620292 28978152

[146] Martinez-Outschoorn UE Goldberg A Lin Z Ko YH Flomenberg N Wang C. Anti-estrogen resistance in breast cancer is induced by the tumor microenvironment and can be overcome by inhibiting mitochondrial function in epithelial cancer cells. Cancer Biol Ther. (2011) 12:924–38. doi: 10.4161/cbt.12.10.17780 PMC3280908 22041887

[147] Sinha S Vegesna R Mukherjee S Kammula AV Dhruba SR Wu W. PERCEPTION predicts patient response and resistance to treatment using single-cell transcriptomics of their tumors. Nat Cancer. (2024) 5:938–52. doi: 10.1038/s43018-024-00756-7 PMC13447014 38637658

[148] Gonzalez Castro LN Tirosh I Suvà ML. Decoding cancer biology one cell at a time. Cancer Discovery. (2021) 11:960–70. doi: 10.1158/2159-8290.CD-20-1376 PMC8030694 33811126

[149] Li M Wu L Si H Wu Y Liu Y Zeng Y. Engineered mitochondria in diseases: mechanisms, strategies, and applications. Signal Transduct Target Ther. (2025) 10:71. doi: 10.1038/s41392-024-02081-y 40025039 PMC11873319

[150] Ross MF Kelso GF Blaikie FH James AM Cochemé HM Filipovska A. Lipophilic triphenylphosphonium cations as tools in mitochondrial bioenergetics and free radical biology. Biochem (Mosc). (2005) 70:222–30. doi: 10.1007/s10541-005-0104-5 15807662

[151] Lopez Sanchez MIG Krüger A Shiriaev DI Liu Y Rorbach J. Human mitoribosome biogenesis and its emerging links to disease. Int J Mol Sci. (2021) 22:3827. doi: 10.3390/ijms22083827 33917098 PMC8067846

[152] Ma Y Zhu S Lv T Gu X Feng H Zhen J. SQSTM1/p62 Controls mtDNA Expression and Participates in Mitochondrial Energetic Adaption via MRPL12. iScience. (2020) 23:101428. doi: 10.1016/j.isci.2020.101428 32805647 PMC7452302

[153] Shayota BJ. Biomarkers of mitochondrial disorders. Neurotherapeutics. (2024) 21:e00325. doi: 10.1016/j.neurot.2024.e00325 38295557 PMC10903091

[154] Shi Y Shi M Wang Y You J. Progress and prospects of mRNA-based drugs in pre-clinical and clinical applications. Signal Transduct Target Ther. (2024) 9:322. doi: 10.1038/s41392-024-02002-z 39543114 PMC11564800

[155] Altzerinakou MA Collette L Paoletti X. Cumulative toxicity in targeted therapies: what to expect at the recommended phase II dose. J Natl Cancer Inst. (2019) 111:1179–85. doi: 10.1093/jnci/djz024 PMC6855968 30838405

[156] Kim TW Kim B Kim JH Kang S Park SB Jeong G. Nuclear-encoded mitochondrial MTO1 and MRPL41 are regulated in an opposite epigenetic mode based on estrogen receptor status in breast cancer. BMC Cancer. (2013) 13:502. doi: 10.1186/1471-2407-13-502 24160266 PMC4015551

[157] Abaji R Ceppi F Patel S Gagné V Xu CJ Spinella JF. Genetic risk factors for VIPN in childhood acute lymphoblastic leukemia patients identified using whole-exome sequencing. Pharmacogenomics. (2018) 19:1181–93. doi: 10.2217/pgs-2018-0093 30191766

